# Robust differentiation of NK cells from MSLN.CAR-IL-15–engineered human iPSCs with enhanced antitumor efficacy against solid tumors

**DOI:** 10.1126/sciadv.adt9932

**Published:** 2025-05-02

**Authors:** Qun Jiang, Weiming Yu, James Ma, Mingming Zhao, Jizhong Zou, Sameer Mir, Jingli Zhang, Ronald N. Germain, Raffit Hassan

**Affiliations:** ^1^ Thoracic and GI Malignancies Branch, Center for Cancer Research (CCR), National Cancer Institute (NCI), National Institutes of Health (NIH), Bethesda, MD, USA.; ^2^ Lymphocyte Biology Section, Laboratory of Immune System Biology, National Institute of Allergy and Infectious Diseases (NIAID), NIH, Bethesda, MD, USA.; ^3^ Center for Advanced Tissue Imaging, NIAID and NCI, NIH, Bethesda, MD, USA.; ^4^ University of Pittsburgh School of Medicine, Pittsburgh, PA, USA.; ^5^ iPSC Core, National Heart, Lung, and Blood Institute, NIH, Bethesda, MD, USA.

## Abstract

Human induced pluripotent stem cells (iPSCs) offer a promising source for chimeric antigen receptor (CAR)–engineered natural killer (NK) products. However, complex iPSC-NK (iNK) manufacturing challenges clinical use. Here, we identified LiPSC-GR1.1 as a superior iPSC line for iNK production. By engineering LiPSC-GR1.1 with a mesothelin (MSLN)–targeting CAR and interleukin-15 (IL-15), we achieved robust differentiation of iPSCs into mature activated iNK cells with enhanced tumor killing efficacy, superior tumor homing, and vigorous proliferation. Single-cell transcriptomic analysis revealed that transforming growth factor–β (TGF-β)–producing tumor cells up-regulated major histocompatibility complex molecules and down-regulated MSLN post–CAR-IL-15 iNK treatment. Tumor-infiltrating CAR-IL-15 iNK cells exhibited high levels of CAR, IL-15, and NK-activating receptors, negligible checkpoint exhaustion markers, and extremely low levels of NK suppressive factors *CISH*, *TGFBR2*, and *BATF*, enabling them to sustain activation, metabolic fitness, and effective tumor killing within TGF-β–rich hypoxic tumor microenvironment. Overall, we developed MSLN.CAR-IL-15–engineered GR1.1-iNK therapy with enhanced antitumor efficacy for solid tumor treatment.

## Teaser

MSLN.CAR-IL-15 engineering boosted iPSC-NK production and improved iNK antitumor efficacy.

## INTRODUCTION

Chimeric antigen receptor (CAR) T cell therapy has been highly effective for treating blood cancers but shows limited success against solid tumors (*1*). CAR-engineered natural killer (NK) cells provide both CAR-dependent and CAR-independent antitumor effects with lower toxicity and offer scalable off-the-shelf production, making them a promising alternative cell therapy (*2*, *3*). Induced pluripotent stem cell (iPSC)–based NK (iNK) cell therapies have several advantages, such as a nearly limitless source, efficient and stable CAR expression with one-time genetic modification, and a relatively homogenous cell product with a more standardized production pipeline (*4*). A recent clinical report on FT596, an iPSC-derived CD19 CAR NK cell therapy, demonstrated good tolerance and encouraging outcomes in patients with relapsed or refractory B cell lymphoma, achieving an complete response rate of 85% in follicular lymphoma, 64% in aggressive large-cell lymphomas excluding de novo diffuse large B cell lymphoma, and 30% among 20 patients who had previously failed CD19 CAR T cell therapy (*5*). This study supports iPSC-derived, gene-modified NK cells as highly promising allogeneic cell therapy for cancer and other diseases. However, CAR iNK manufacturing is a complex and challenging process that includes CAR engineering in iPSCs, differentiating iPSCs into functional iNK cells and expanding these cells at a large scale (*6*). iPSCs derived from different donors exhibit genetic variability, which can influence the differentiation potential, functionality, and safety profile of the resulting iNK cells (*7*). Thus, iPSC line diversity also poses challenges for standardizing cell manufacturing processes for iPSC-based therapies (*8*). The Regenerative Medicine Program was launched by the US National Institutes of Health (NIH) to develop resources to catalyze therapeutic use of iPSCs. To date, this program has generated 1 clinical-grade current Good Manufacturing Practices (cGMP) iPSC line and 14 research-grade iPSC lines, all of which are accessible to the research community. Among them, six iPSC lines were generated from healthy donor–derived cord blood CD34^+^ cells, including the cGMP-manufactured standardized line LiPSC-GR1.1, which has been well characterized (*9*, *10*). Ideally, using well-characterized cGMP-manufactured human iPSC lines to produce CAR iNK products may result in time and cost reduction in translation into the clinic because of elimination of the time-consuming and resource-intensive quality control measures needed for characterization of newly reprogrammed iPSCs.

Mesothelin (MSLN) is a glycosylphosphatidylinositol-anchored membrane glycoprotein highly expressed in many solid tumors but with limited expression on normal tissues, making it an attractive target antigen for a cell therapy (*11*). We previously generated a highly effective CAR T product using the sequence of humanized hYP218 scFv targeting the MSLN membrane–proximal region. This CAR T product showed superior tumor killing and intratumoral infiltration as compared to CAR T generated using the SS1 scFv targeting the MSLN membrane–distal region (*12*). Thus, we hypothesized that hYP218 scFv would also be promising for creating iPSC-derived CAR NK for treatment of MSLN-positive solid tumors. A very recent study also showed that YP218.CAR-engineered NK derived from peripheral blood (PBNK) had superior antitumor efficacy as compared to SS1.CAR-engineered PBNK in MSLN-positive ovarian cancer (*13*).

NK cells generally have a short lifespan in vivo, especially without support from cytokines like interleukin (IL-2) or IL-15 (*14*), which plays a critical role in NK cell development and homeostasis (*15*, *16*). IL-15 has been included in CAR constructs for genetically modifying primary NK products to locally enhance NK cell survival, expansion, and antitumor activity (*17*). Most of the research in this area has focused on hematopoietic cancers, such as IL-15–expressing CD19 CAR NK therapies (*18*–*20*). Studies investigating IL-15–expressing CAR NK cells in solid tumors are still relatively limited, and even fewer have explored the use of IL-15-CAR–engineered iPSC-NK in this setting. Consequently, our understanding of the molecular features of IL-15–expressing CAR iNK in the context of local solid tumors remains unclear.

In this study, we identified LiPSC-GR1.1 as a superior iPSC line for iNK production. We genetically engineered LiPSC-GR1.1 using a nonviral PiggyBac transposon system expressing MSLN-targeting hYP218.CAR plus IL-15 and achieved robust differentiation of MSLN.CAR-IL-15 GR1.1-iNK cells. We then explored NK biomarker and transcriptional profiles of the GR1.1-iNK product and evaluated the tumor killing efficacy of the GR1.1-iNK cells using in vitro and animal model studies. In addition, we demonstrated tumor infiltration and proliferation of the MSLN.CAR-IL-15 GR1.1-iNK cells in a xenograft tumor model and investigated the transcriptional signatures of iNK-treated tumor as well as tumor-infiltrating MSLN.CAR-IL-15 GR1.1-iNK cells using single-cell sequencing.

## RESULTS

### LiPSC-GR1.1 identified as a superior human iPSC line for NK cell differentiation

**Fig. 1.**
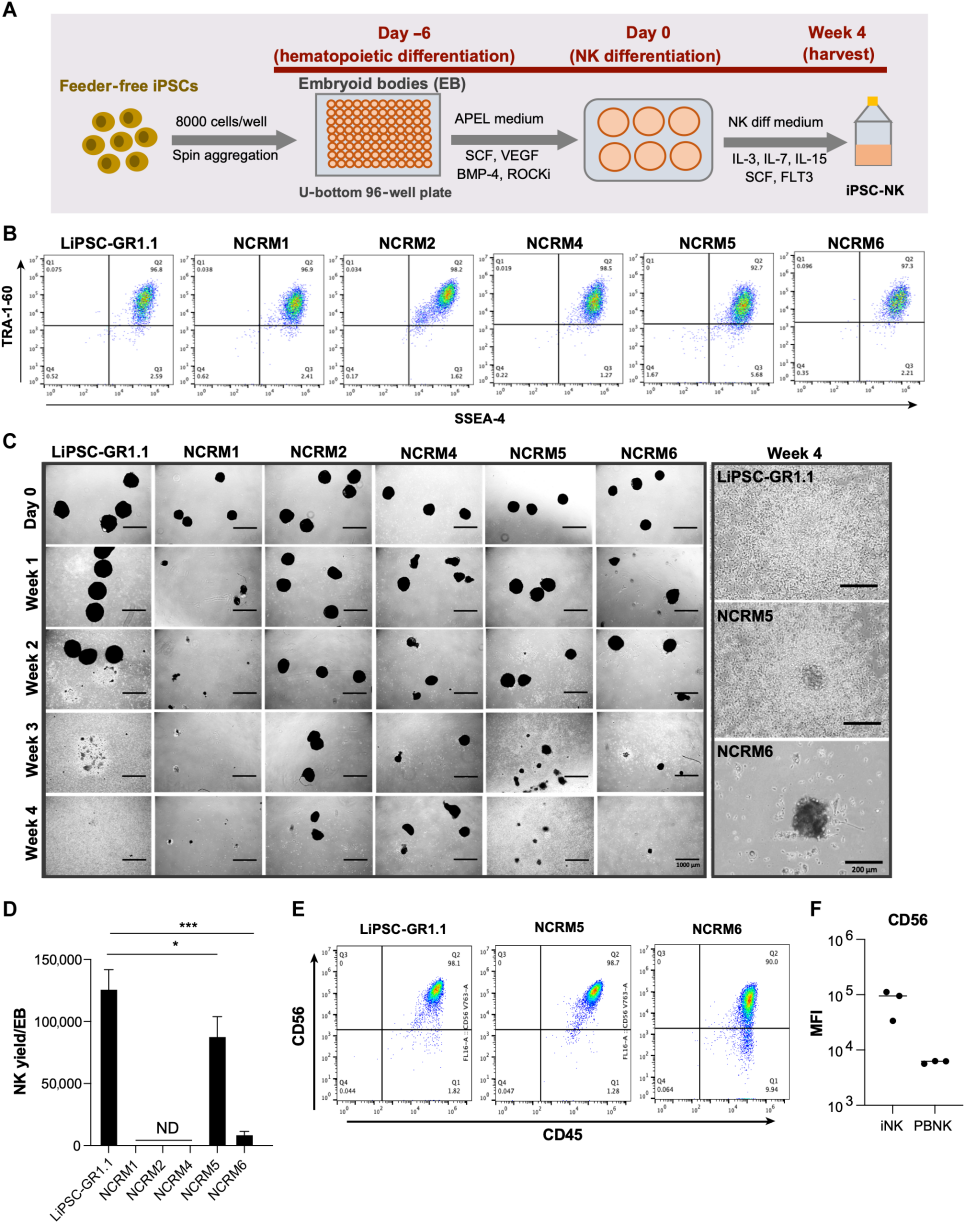
Differentiation of iNK cells from six human iPSC lines. (**A**) Scheme of NK differentiation from iPSCs. EBs were generated using the spin method from a single-cell dissociated human iPSCs seeded on day −6 and maintained under feeder- and serum-free condition in a U-bottom 96-well plate. Six days later (after hematopoietic progenitor cells generated), EBs were transferred into NK cell differentiation conditions on day 0. NK cells were then harvested 4 weeks postdifferentiation. (**B**) The expression of pluripotency markers SSEA-4 and TRA-1-60 was detected in the iPSC lines via flow cytometry. (**C**) Left panel: morphology of day 0 EBs and 4 weeks of NK differentiation from six different iPSC cell lines. Scale bars represent 1000 μm. Right panel: representative magnified pictures of differentiated iNK cells 4 weeks post-NK differentiation. Scale bars represent 200 μm. (**D**) iNK differentiation yield per EB from different iPSC lines (*n* = 2 to 3 independent differentiation experiments performed for each iPSC line). ND means that no differentiation was observed in these lines. *P < 0.05 and ***P < 0.001, analyzed using unpaired Student’s *t* test. (**E**) NK differentiation from the three iPSC lines LiPSC-GR1.1, NCRM5, and NCRM6 was validated by staining for CD45 and CD56. (**F**) The median fluorescence intensity (MFI) of CD56 was measured by flow cytometry on PBNK cells (from three healthy donors) and iNK cells (derived from LiPSC-GR1.1, NCRM5, and NCRM6).

We selected the well-characterized LiPSC-GR1.1 line (*9*, *10*) and five NIH Center for Regenerative Medicine (NCRM) iPSC lines with a normal karyotype for NK differentiation study. The NCRM iPSC lines exhibited a similar SSEA-4^+^TRA-1-60^+^ pluripotency phenotype (Fig. 1B) and were validated for their trilineage differentiation potential through scorecard assay (fig. S1). Using a standardized, simple, and efficient “spin embryoid body (EB)” iPSC-NK differentiation protocol (Fig. 1A) (*21*), we compared LiPSC-GR1.1 to the five NCRM iPSC lines for their iNK differentiation potential. A total of 8000 TrypLE-adapted iPSCs was seeded in each well of 96-well round-bottom plates in APEL culture medium containing a Rho-associated coiled-coil kinase (ROCK) inhibitor, stem cell factor (SCF), vascular endothelial growth factor (VEGF), and bone morphogenetic protein 4 (BMP-4) and spun to form EBs. All the cell lines initially aggregated and formed EBs on day 6 postcell seeding (Fig. 1C). After day 6, EBs were then directly transferred into six-well plates with NK cell differentiation medium for 4 weeks. During weeks 2 to 4, we observed substantial hematopoietic cell differentiation from EBs derived from LiPSC-GR1.1 and NCRM5. The EBs of these two lines began to disappear, with more free-floating, multicellular aggregates observed during the third week of NK differentiation. A low number of floating hematopoietic cells were observed with the EBs derived from the NCRM6 line. The EBs derived from NCRM1, NCRM2, and NCRM4 failed to further differentiate. The average iNK differentiation yields were 1.3 × 10^5^ per EB, 8.8 × 10^4^ per EB, and 9.0 × 10^3^ per EB from iPSC lines LiPSC-GR1.1, NCRM5, and NCRM6, respectively (Fig. 1D). After 4 weeks of NK differentiation, >98% of cells harvested from the supernatant of LiPSC-GR1.1 and NCRM5, as well as >90% of cells harvested from the supernatant of NCRM6, were CD45^+^CD56^+^ NK cells (Fig. 1E) with higher CD56 expression as compared to the PBNK cells (Fig. 1F).

### Robust differentiation of NK cells from MSLN.CAR-IL-15–engineered LiPSC-GR1.1 cells

**Fig. 2.**
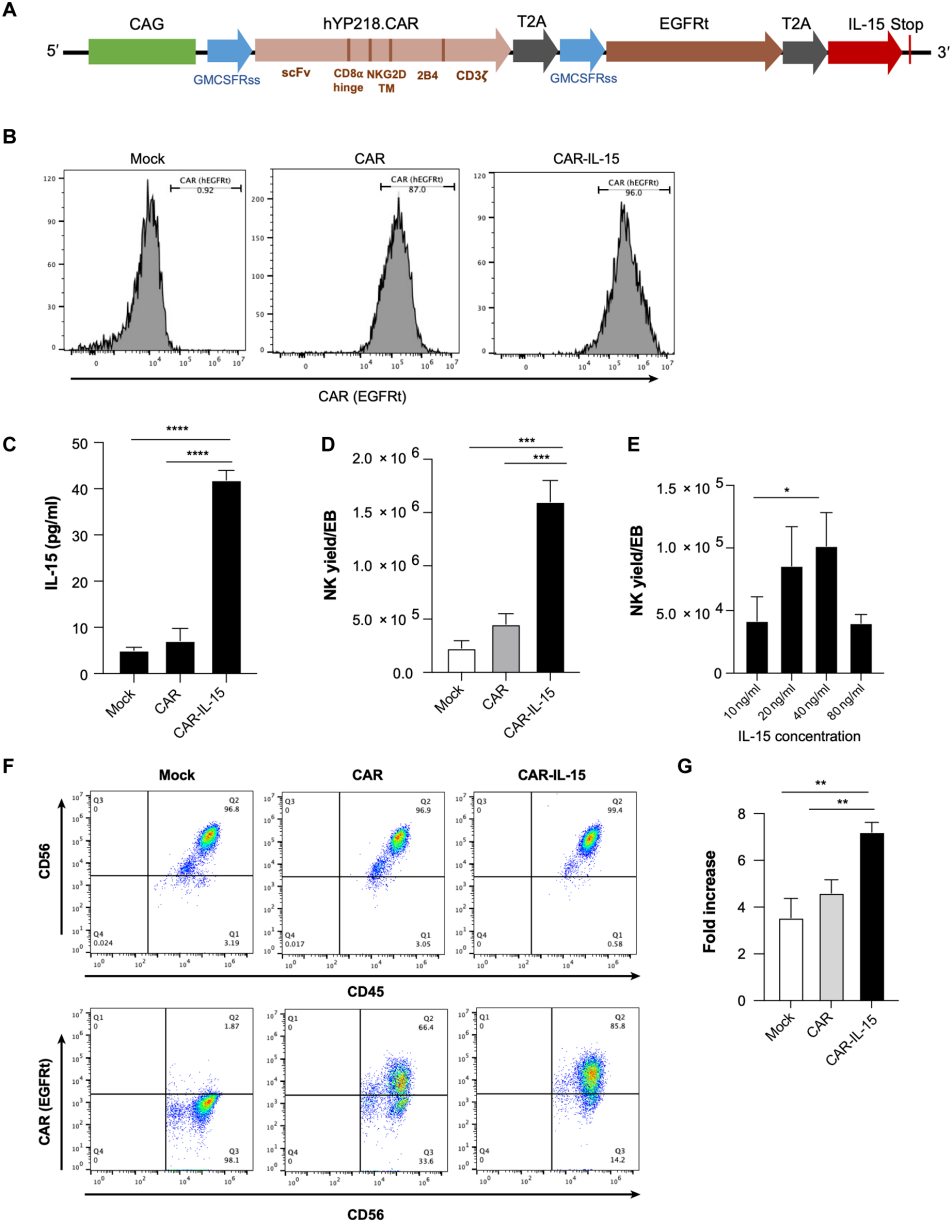
Robust iNK differentiation yield from MSLN.CAR-IL-15–transduced LiPSC-GR1.1. (**A**) MSLN.CAR-IL-15 piggyBac transposon vector construct encoding the CAG promoter, MSLN.CAR (consisted of MSLN-targeted hYP218 scFv, CD8a hinge spacer, NKG2D transmembrane domain, 2B4 ICD, and CD3ζ chain stimulatory domain), EGFRt, and human IL-15. GMCSFRss, GM-CSF receptor-α chain signal sequence directing cell surface expression. (**B**) Genetic modification of LiPSC-GR1.1 cells with MSLN.CAR and MSLN.CAR-IL-15 piggyBac transposon systems. MSLN.CAR expression was examined by measuring EGFRt expression in transfected iPSCs. (**C**) Human IL-15 production in the supernatant of iPSC culture. ****P < 0.0001. (**D**) NK differentiation yield per EB from MSLN.CAR-IL-15–modified iPSCs and control iPSCs. ***P < 0.001. (**E**) iNK differentiation yield achieved with different concentrations of IL-15 (10, 20, 40, and 80 ng/ml) added to the mock LiPSC-GR1.1-iNK differentiation media. *P < 0.05. (**F**) NK differentiation and CAR expression in NK were validated by detecting CD45, CD56, and EGFRt expression in the cells. (**G**) Cryopreserved differentiated iNK cells were thawed and expanded for 7 days. The fold increase of expansion is shown. **P < 0.01. The statistics were analyzed using unpaired Student’s *t* test.

To target MSLN-expressing solid tumors, the DNA construct MSLN.CAR that encodes the hYP218 scFv, followed by a CD8a hinge spacer, the NK group 2 member D (NKG2D) transmembrane domain (TM), the 2B4 intracellular domain (ICD), and the CD3ζ intracellular signaling domain, along with an independently translated truncated human epidermal growth factor receptor (EGFR) polypeptide (EGFRt) sequence for tracking purpose, was subcloned into a PiggyBac transposon vector. IL-15 was also included in the construct to support NK persistence in vivo (Fig. 2A) (*9*). We then genetically engineered LiPSC-GR1.1 cells with the PiggyBac transposon system carrying MSLN.CAR or MSLN.CAR-IL-15 and enriched the iPSCs stably expressing the components of the constructs by fluorescence-activated cell sorting (FACS) of EGFRt^+^ iPSCs. CAR expression by the expanded CAR or CAR-IL-15–engineered LiPSC-GR1.1 cells was validated using flow cytometry (Fig. 2B). IL-15 production was detected only in the supernatant of the cultures of CAR-IL-15–engineered iPSCs using enzyme-linked immunosorbent assay (ELISA) (Fig. 2C). We next tested the NK cell differentiation potential of these iPSCs. It is observed that a robust yield of iNK cells was attained from MSLN.CAR-IL-15 iPSCs (~1.6 × 10^6^ per EB) compared to the limited iNK yield from mock iPSCs (~2.3 × 10^5^ per EB) or MSLN.CAR-engineered iPSCs (~4.5 × 10^5^ per EB) at 4 weeks postdifferentiation initiation (Fig. 2D and fig. S2). In contrast, we only observed a slightly increased NK cell yield from the LiPSC-GR1.1 cells when we increased the IL-15 concentrations to 20 and 40 ng/ml in the differentiation medium (Fig. 2E), suggesting that genetically modifying iPSCs to constantly express endogenous IL-15 ensures more robust and sustained signaling for hematopoietic differentiation within the mesoderm. We then examined NK markers and CAR expression by these differentiated iNK cells using flow cytometry. The percentage of CD45^+^CD56^+^ NK cells in culture supernatants was >96% in all three groups (Fig. 2F). CAR expression was 66.4% in MSLN.CAR iNK cells and 85.8% in MSLN.CAR-IL-15 iNK cells. In addition to the significant increase in differentiation yield, the recovery and expansion capacity of differentiated MSLN.CAR-IL-15 iNK cells post–cryopreservation was also observed to be superior compared to both mock iNK cells and MSLN.CAR iNK cells (Fig. 2G), suggesting that IL-15 expression could also benefit the viability and functionality of iNK cells after preservation.

### Phenotypic and transcriptome profiling of MSLN.CAR-IL-15–engineered GR1.1-iNK cells

**Fig. 3.**
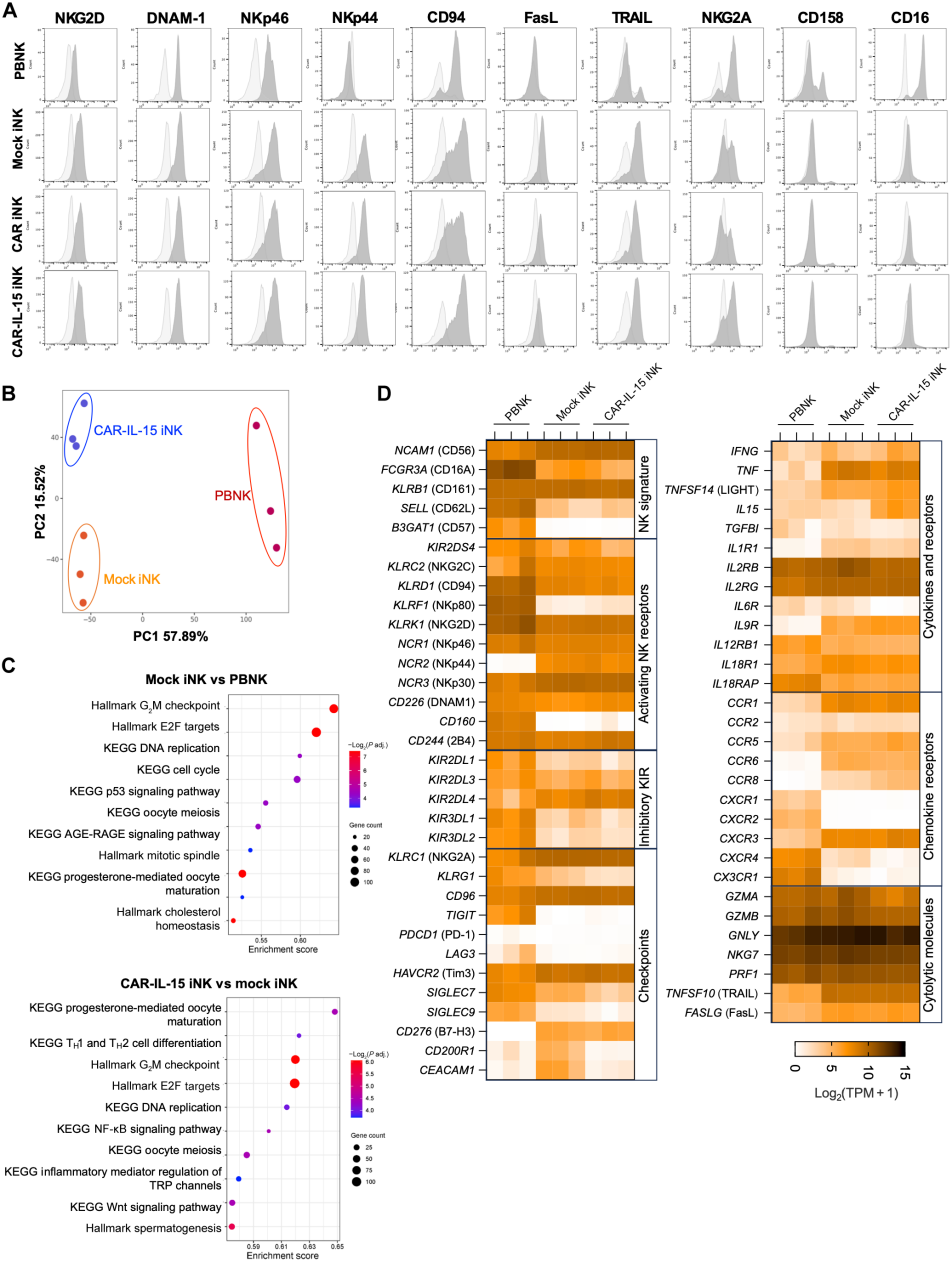
Characterization of freshly differentiated GR1.1-iNK cells. GR1.1-iNK cells were harvested 4 weeks post-EB differentiation. (**A**) The cell surface receptor expression in NK cells was detected via flow cytometry. CD56^+^CD3^−^ PBNK and CD56^+^ GR1.1-iNK cells were gated for the indicated biomarker analyses. The dark gray area represents biomarker staining. The light gray area represents isotype control. (**B** to **D**) RNA-seq of PBNK, mock iNK, and MSLN.CAR-IL-15 iNK cells. Data are from one RNA-seq analysis with three biological replicates per group. (B) PCA of DEGs. Each dot represents one sample. Each color represents an NK population. (C) Bubble plot of GSEA analysis showing the highest-ranked top 10 KEGG and Hallmark gene sets significantly up-regulated (*P* < 0.05, adjusted *P* < 0.1) in mock iNK versus PBNK (top panel) and MSLN.CAR-IL-15 iNK cells versus mock iNK cells (bottom panel). The *x* axis represents the enrichment score, and the size of the bubble represents the number of genes in the gene set. (D) The heatmap shows the gene expression [log_2_(TPM + 1)] (where TPM means transcripts per million) of NK phenotypic markers in PBNK, mock iNK, and MSLN.CAR-IL-15 iNK cells, including NK signature, activating receptors, inhibitory KIR, checkpoint, cytokine and receptors, chemokine receptors, and cytolytic molecule–related genes.

We then used flow cytometry to detect NK biomarkers on iNK cells freshly harvested at 4 weeks post–initiation of differentiation, as compared to the PBNK control. We observed high levels of NKG2D, DNAM-1, NKp46, NKp44, CD94, Fas-L, and TRAIL, intermediate level of NKG2A, and low levels of CD16 and CD158 (KIR2DL1/S1/S3/S5) on mock iNK, MSLN.CAR iNK, and MSLN.CAR-IL-15 iNK cells (Fig. 3A). To compare transcriptional profiling of freshly differentiated iNK cells to mature PBNK cells, we performed RNA sequencing (RNA-seq) on the RNA extracted from healthy donor PBNK, mock iNK, and MSLN.CAR-IL-15–modified iNK cells. Principal components analysis (PCA) (Fig. 3B) and unsupervised hierarchical clustering of gene expression (fig. S3A) showed that three NK populations formed three distinct clusters. PBNK cells were strongly separated from mock iNK and MSLN.CAR-IL-15 iNK cells along the PC1 axis; mock iNK and MSLN.CAR-IL-15 iNK cells shared a similar PC1 value but were clearly separated along the PC2 axis. We then analyzed the differentially expressed genes (DEGs) and the corresponding gene-enriched pathways. Overall, 4724 DEGs (2373 up-regulated and 2351 down-regulated) were identified when comparing mock iNK cells versus PBNK cells; 1371 DEGs (649 up-regulated and 722 down-regulated) were identified when comparing MSLN.CAR-IL-15 iNK cells versus mock iNK cells (fig. S3B). Kyoto Encyclopedia of Genes and Genomes (KEGG) and Hallmark pathway gene set enrichment analysis (GSEA) of the DEGs showed a significant increase in pathways including cell division, DNA replication, p53 signaling pathway, AGE-RAGE (advanced glycation end product-receptor for advanced glycation end product) signaling pathway, and cholesterol homeostasis in iNK cells as compared to PBNK cells (Fig. 3C). Compared to mock iNK cells, MSLN.CAR-IL-15 iNK cells showed increased expression of genes associated with cell division, DNA replication, nuclear factor κB (NF-κB) signaling, transient receptor potential channel calcium signaling, and Wnt signaling pathways. We then profiled the expression of NK activity–associated genes in different categories (Fig. 3D). Although GR1.1-iNK cells showed similar expression of many NK markers in common with PBNK cells, they exhibited distinct features. The genes that were increased in GR1.1-iNK cells included *NCAM1* (CD56), *NCR2* (NKp44), *NCR3* (NKp30), *KLRC1* (NKG2A), *CD96*, *HAVCR2* (Tim3), *CD276* (B7-H3), *TNF*, *TNFSF14* (LIGHT), *CCR1*, *CCR5*, C*CR6*, *CCR8*, *CXCR3*, and *TNFSF10* (TRAIL), while the genes that were decreased in GR1.1-iNK cells included *FCGR3A* (CD16A), *B3GAT1* (CD57) *KLRF1* (NKp80), *CD160, TIGIT*, *LAG3, CXCR1*, *CXCR2*, *CXCR4*, and *CX3CR1*. MSLN.CAR-IL-15 iNK cells expressed a similar level of most markers and cytokines, except for higher *IL15* and lower *CD200R1* and *CEACAM1*, as compared to mock iNK cells. In addition, we observed a high level expression of some “adaptive NK” feature genes *IL32*, *S100A4*, and *ITGA1* (CD49A) in GR1.1-iNK cells (fig. S3C) (*22*).

### Enhanced cytotoxicity of MSLN.CAR-IL-15 iNK cells against human solid tumor cells

**Fig. 4.**
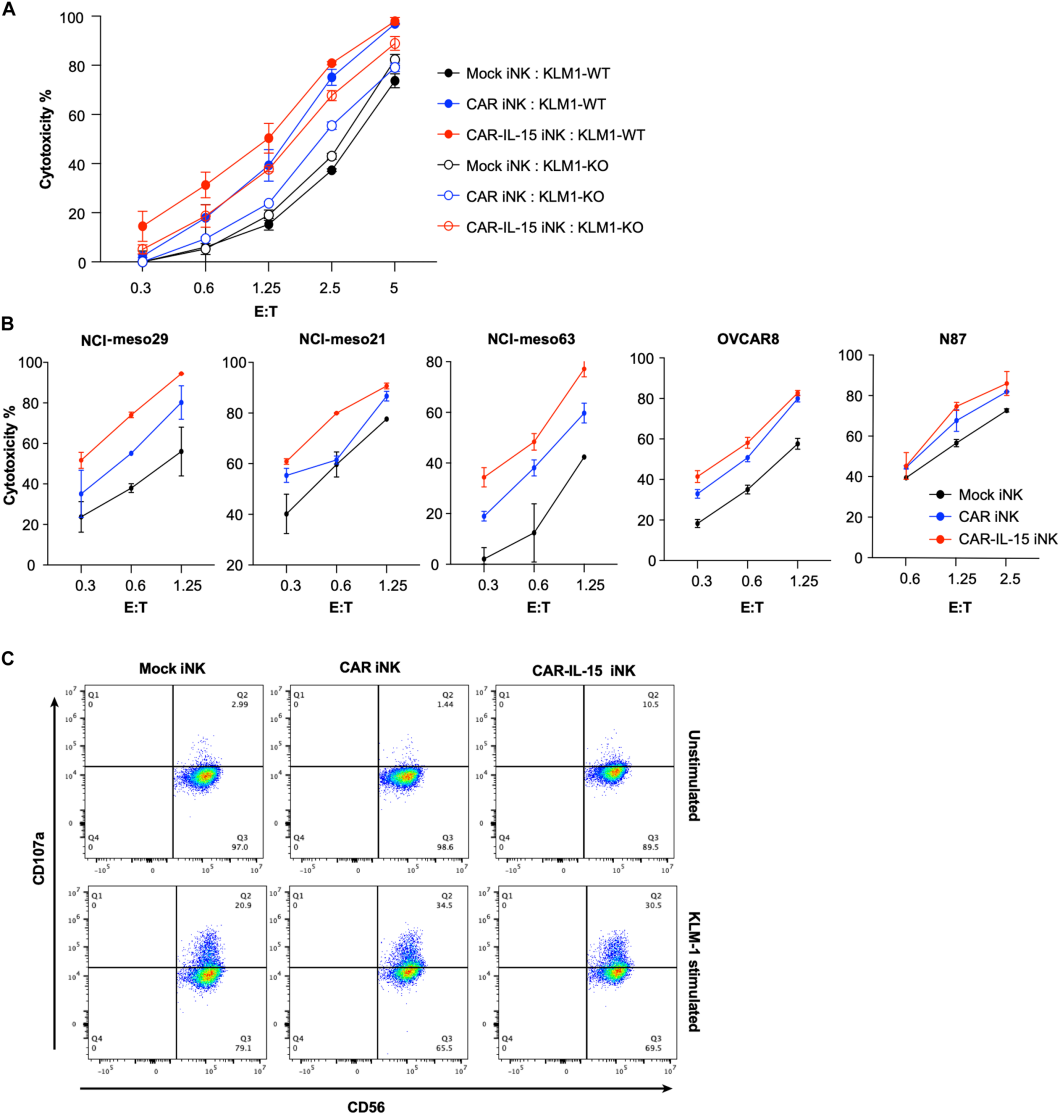
In vitro tumor killing function of MSLN.CAR-IL-15 GR1.1-iNK cells. (**A**) Cytotoxicity of mock iNK, MSLN.CAR iNK, and MSLN.CAR-IL-15 iNK cells against MSLN^+^ KLM1-WT and MSLN^−^ KLM1-KO cells. (**B**) Cytotoxicity of all the iNK cells against multiple MSLN^+^ solid tumor cell lines. (**C**) CD107a degranulation signature induced by the coculture of iNK cells with KLM-1 tumor cells.

To evaluate the tumor cell killing ability of the GR1.1-iNK cells, we performed cytotoxicity assays by coculturing different modified GR1.1-iNK cells with target cancer cell lines for 24 hours. MSLN.CAR-IL-15 iNK displayed the highest killing ability against all the tumor cells we tested (pancreatic cancer KLM-1, mesothelioma NCI-meso29, NCI-meso21, NCI-meso63, ovarian cancer OVCAR8, and gastric carcinoma cell line NCI-N87), as compared to mock iNK and MSLN.CAR iNK (Fig. 4, A and B). MSLN.CAR iNK and MSLN.CAR-IL-15 iNK killed more MSLN^+^ KLM1-WT than MSLN^−^ KLM1-KO target cells, suggesting that MSLN recognition by CAR increased specific killing (Fig. 4A). On the other hand, MSLN.CAR-IL-15 iNK showed a higher killing ability than mock iNK and MSLN.CAR iNK when cocultured with MSLN^−^ KLM1-KO target cells, indicating that chronic stimulation by IL-15 led to NK activation that promoted cytotoxicity independent of the CAR targeting specificity (Fig. 4A). We then tested CD107a expression (granule release) of iNK cells. Without target stimulation, we observed slightly higher CD107a expression in MSLN.CAR-IL-15 iNK cells than in mock iNK and MSLN.CAR iNK cells; when stimulated with KLM-1 tumor cells, MSLN.CAR iNK and MSLN.CAR-IL-15 iNK cells demonstrated higher CD107a expression, while mock iNK cells had limited CD107a expression (Fig. 4C). These results demonstrate that MSLN.CAR-IL15 modification of the LiPSC-GR1.1 cells not only significantly increased the iNK differentiation yield but also markedly enhanced the killing function of the differentiated iNK against various tumor cells.

### Tumor regression and improved survival in MSLN.CAR-IL15 iNK–treated MSLN^+^ tumor–bearing mice

**Fig. 5.**
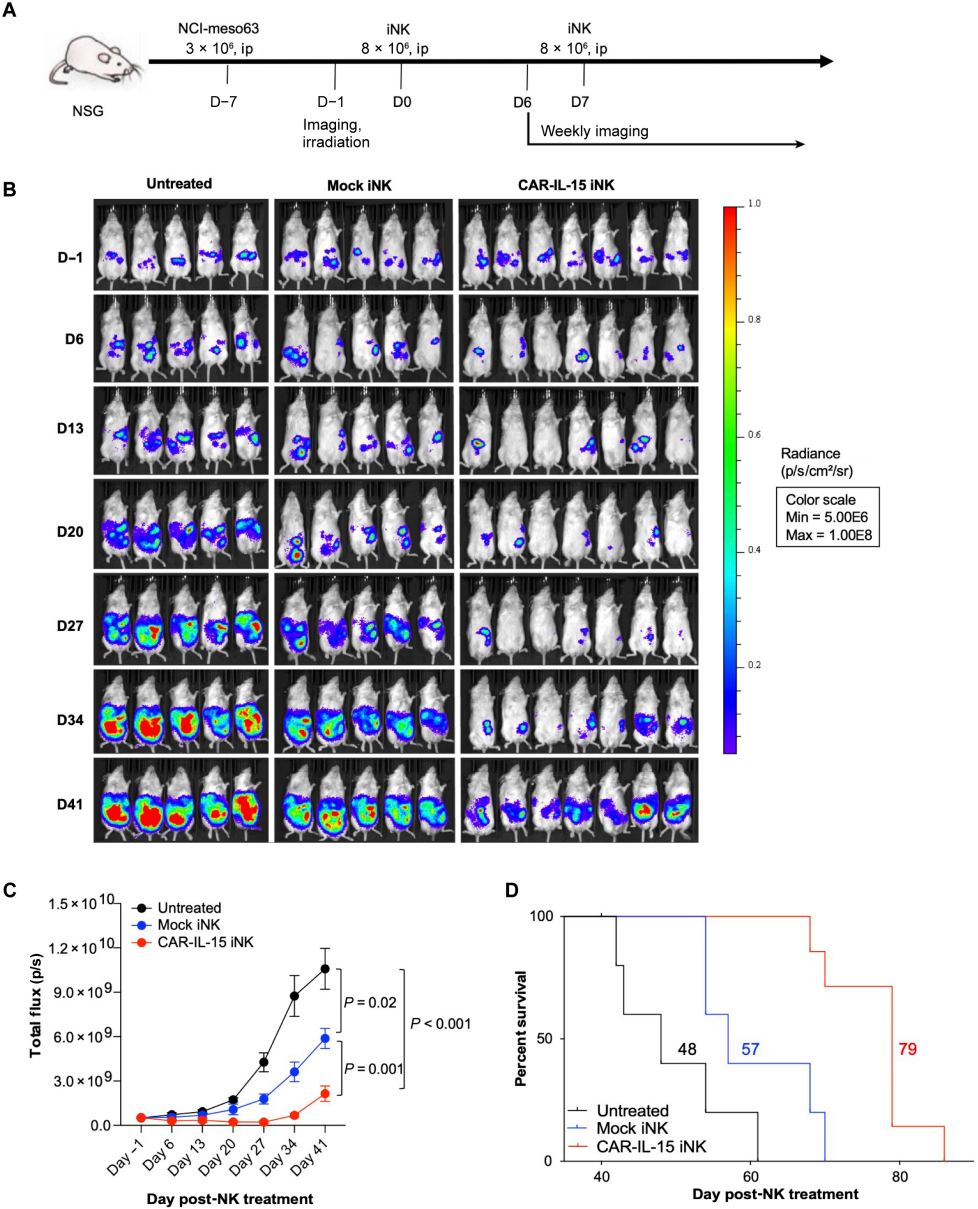
Antitumor efficacy of MSLN.CAR-IL-15 GR1.1-iNK cells in the NCI-meso63 mouse model. (**A**) Schematic of tumor inoculation and iNK treatments in the NCI-meso63 tumor model. ip, intraperitoneally; D, day. (**B**) Tumor growth monitored through BLI. *P* values were analyzed using unpaired Student’s *t* test. (**C**) Tumor growth based on BLI measurements. The statistics were analyzed using unpaired Student’s *t* test. (**D**) The median overall survival of different groups is shown [*P* = 0.07, mock iNK versus untreated; *P* < 0.005, CAR-IL-15 iNK versus mock iNK, analyzed using the log-rank (Mantel-Cox) test].

To evaluate the antitumor efficacy of MSLN.CAR-IL15 iNK cells in vivo, we first used the patient mesothelioma-derived xenograft NCI-meso63 mouse model established previously (*23*). The luciferase-expressing NCI-meso63 tumor cells were injected intraperitoneally 7 days before iNK treatment. The mice were then administered two doses of freshly differentiated iNK cells on days 0 and 7 and monitored weekly using bioluminescence imaging (BLI) (Fig. 5A). Tumor growth was markedly decreased in mice receiving MSLN.CAR-IL-15 iNK cells as compared to mice receiving mock iNK cells, although the mice receiving mock iNK cells did show some antitumor effects when compared to the untreated animals because of the CAR-independent activity of NK cells (Fig. 5, B and C). The median overall survival was 48 days without treatment, 57 days with mock iNK treatment, and 79 days with MSLN.CAR-IL-15 iNK treatment (Fig. 5D). In addition, we also tested the iNK in a more aggressive pancreatic tumor KLM-1 mouse model (fig. S4A). Only MSLN.CAR-IL-15 iNK treatment showed significant antitumor effects and prolonged survival, while mock iNK treatment had a minimal effect in this tumor model (fig. S4, B to D). In both mouse models we tested, we did not observe any toxicity in the mice treated with CAR-IL-15 GR1.1-iNK cells. Considering the logistical advantages of cryopreservation for large-scale production and distribution of an off-the-shelf iNK product, we also evaluated the efficacy of iNK expanded from cryopreserved differentiated iNK stocks using the NCI-meso63 tumor model (fig. S5, A to C). These expanded MSLN.CAR-IL-15 iNK cells also exhibited enhanced tumor killing and improved overall survival of these mice compared to the expanded mock iNK treatment. The antitumor activity was similar to the antitumor effects achieved with freshly differentiated MSLN.CAR-IL-15 iNK cells. Given the advantages of scalability and logistical ease, we used the expanded iNK cells from frozen stocks for our following mechanistic mouse model studies.

### Increased infiltration of MSLN.CAR-IL-15 iNK cells in NCI-meso63 tumors

**Fig. 6.**
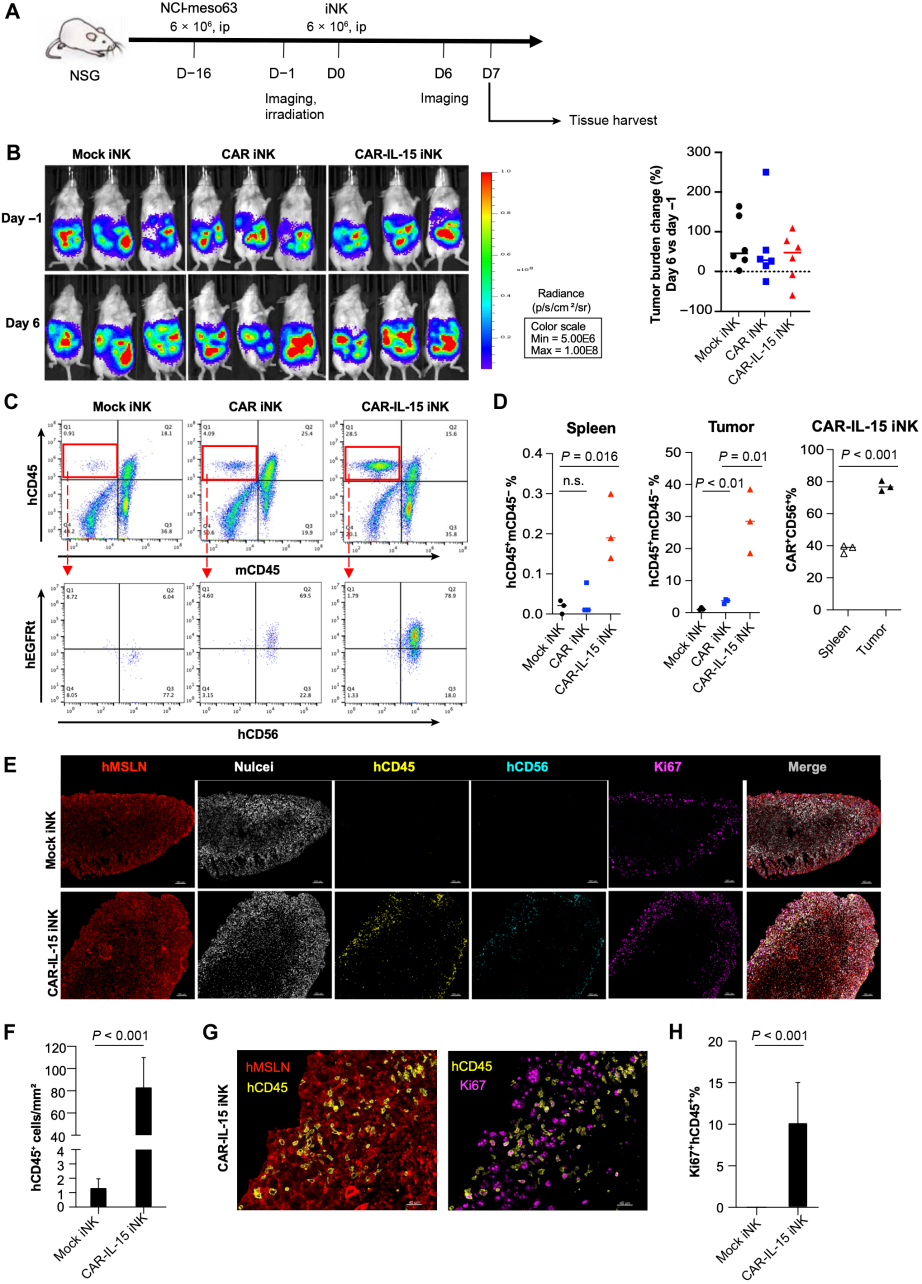
Increased tumor infiltration of MSLN.CAR-IL-15 iNK cells in NCI-meso63 tumor. (**A**) Schematic of iNK treatments and tissue harvest in the NCI-meso63 tumor model. (**B**) Tumor growth monitored using BLI. (**C**) Flow cytometric analysis of hCD45^+^mCD45^−^ cells in the tumors on day 7 post-iNK treatment (top panel). The hCD45^+^mCD45^−^ cells were further gated to analyze hCD56 and hEGFRt expression (bottom panel). (**D**) Percentage of hCD45^+^mCD45^−^ cells among total live single cells isolated from spleens and tumors on day 7 (left panels); percentage of hEGFRt^+^hCD56^+^ cells among hCD45^+^mCD45^−^ cells in spleens and tumors harvested from MSLN.CAR-IL-15 iNK–treated mice on day 7 (right panel). n.s., not significant. (**E**) Multiplex immunofluorescence imaging of tumors harvested from mice treated with mock iNK and MSLN.CAR-IL-15 iNK cells on day 7 post-iNK treatment. Scale bars represent 150 μm. (**F**) hCD45^+^ cell density in iNK-treated tumor. Three independent whole tissue images were analyzed. (**G**) High-magnification images showing Ki67^+^ hCD45^+^ NK cells colocalized with hMSLN^+^ tumor cells in mice treated with MSLN.CAR-IL-15 iNK. Scale bars represent 40 μm. (**H**) Percentage of Ki67^+^ cells among total hCD45^+^ cells in iNK-treated tumor. Three independent whole tissue images were analyzed. *P* values were analyzed using unpaired Student’s *t* test.

Treatment of solid tumors is partially constrained by the limited tumor homing and persistence of NK cells. To investigate the solid tumor infiltration ability of iNK cells, we adjusted the experimental design by increasing the NCI-meso63 tumor cell number, treating the tumor with a lower number of iNK cells, and doing the iNK transfer on day 16 posttumor inoculation when the tumor was substantially larger than in the preceding experiment (Fig. 6A). With only one low-dose iNK treatment, we did not observe significant tumor reduction on day 6 post-iNK treatment (Fig. 6B). We harvested tumor tissue on day 7 after iNK treatment and observed a substantial increase in hCD45^+^hCD56^+^mCD45^−^ cells in the MSLN.CAR-IL-15 iNK–treated tumors compared to tumors from mock iNK–treated mice and CAR iNK–treated mice (Fig. 6, C and D). Approximately 80% of these hCD45^+^hCD56^+^ cells expressed the MSLN.CAR. There was a small number of hCD45^+^mCD45^−^ cells in the spleens of mice given MSLN.CAR-IL-15 iNK cells, and this small number was higher than what was observed in spleens from mock iNK–treated mice (Fig. 6D). Notably, the percentage of CAR^+^ iNK cells was much higher in tumor than in the spleen. We next visualized iNK tumor infiltration in situ using multiplex immunofluorescence staining for MSLN (tumor marker), hCD45/hCD56 (NK marker), and Ki67 (proliferation marker). hCD45^+^ and hCD56^+^ cells were significantly increased in the MSLN.CAR-IL-15 iNK–treated MSLN^+^ tumor as compared to tumors from mice receiving mock iNK cells (Fig. 6, E and F). hCD45^+^ cells colocalized with MSLN^+^ tumor cells, suggesting possible direct interaction (Fig. 6G). Some of these hCD45^+^ cells coexpressed Ki67 (Fig. 6, G and H), indicating recent or ongoing proliferation of MSLN.CAR-IL-15 iNK cells within the tumors. These findings suggest that the MSLN.CAR-IL-15 modification significantly improved iNK cell tumor infiltration and persistence.

### Single-cell transcriptional profiling of MSLN.CAR-IL-15 iNK–treated NCI-meso63 tumors

**Fig. 7.**
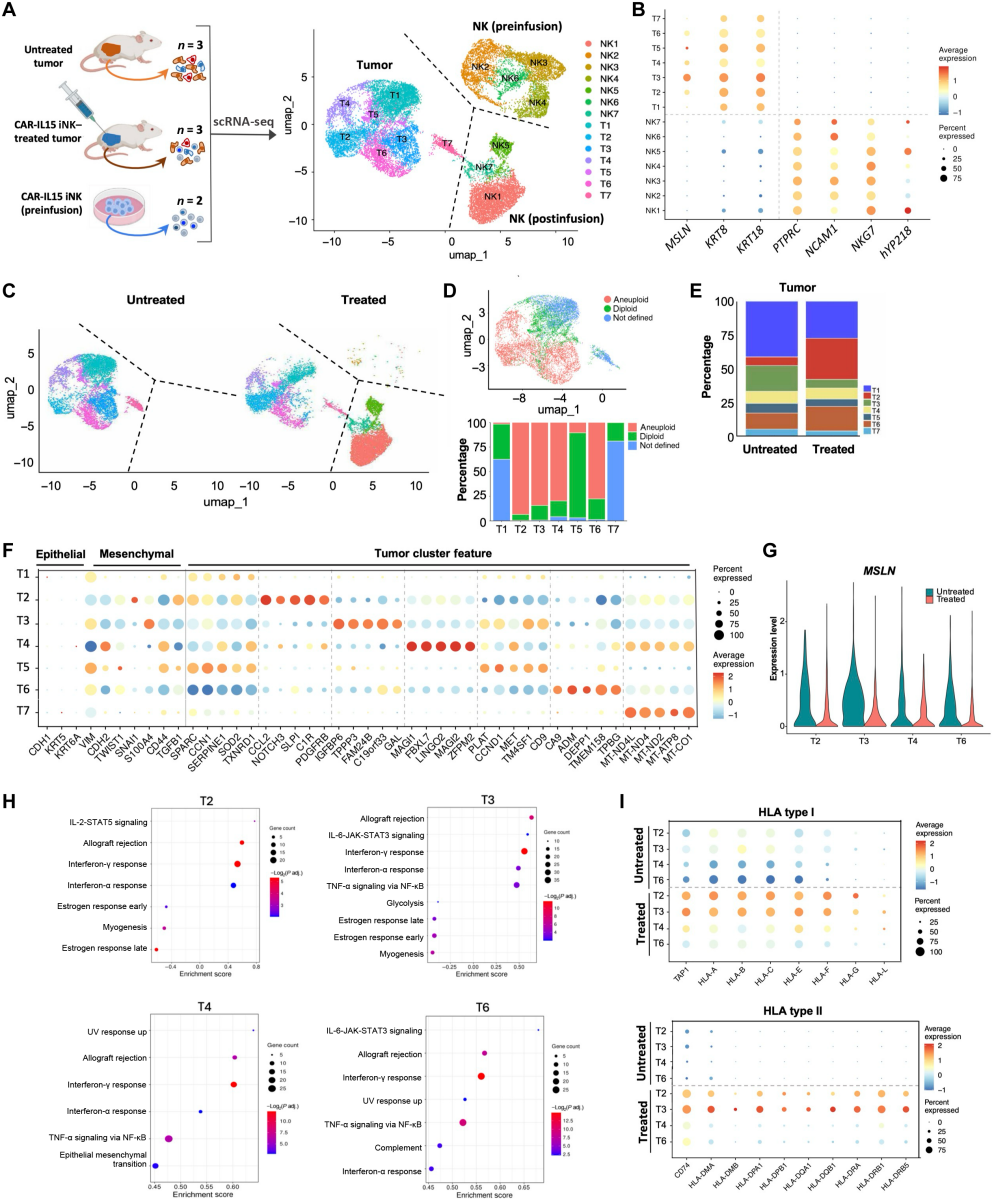
scRNA-seq analysis of MSLN.CAR-IL-15 iNK cell–treated NCI-meso63 tumors. (**A**) UMAP plots visualizing transcriptome-defined clusters of NK cells and tumor cells. (**B**) Representative signature genes across NK and tumor cells. The size of the dots indicates the percent of cells expressing the gene, while the color of the dots indicates the average gene expression level. (**C**) UMAP for total cells from untreated and treated groups. Cell types are annotated with the same color scheme as in (A). (**D**) Fractions of defined tumor subclusters among tumor cells in treated and untreated groups. (**E**) UMAP of identified aneuploid and diploid cells using copyKat analysis (top) and their fractions in each tumor cluster (bottom). (**F**) Expression of representative tumor signature genes across tumor subclusters. (**G**) *MSLN* expression in tumor subclusters in untreated and treated groups. (**H**) Top up-regulated and down-regulated Hallmark pathways in treated versus untreated tumor subclusters (adjusted *P* < 0.25). The *x* axis represents the enrichment scores, and the size of the bubble represents the number of genes in the gene set. JAK, Janus kinase; UV, ultraviolet. (**I**) Expression of HLA type I and HLA type II genes in untreated and iNK-treated tumor subclusters.

NCI-meso63 is a highly metastatic patient mesothelioma-derived xenograft cell line established without single-cell cloning, which retained its heterogeneity (*23*). To characterize the transcriptional state of the NCI-meso63 tumors during therapy with MSLN.CAR-IL-15 iNK cells, as well as that of tumor-infiltrating MSLN.CAR-IL-15 iNK cells, we performed single-cell RNA-seq (scRNA-seq) on total cells isolated from untreated tumors (three samples), iNK-treated tumors (three samples) (same harvest on day 7 post–MSLN.CAR-IL-15 GR1.1-iNK treatment as shown in Fig. 6A), and MSLN.CAR-IL-15 GR1.1-iNK preinfusion product (two samples) (Fig. 7A). To achieve an unbiased comparison among groups, scRNA-seq data from the three groups were pooled for downstream analyses. After quality control filtering, we collected a total of 23,287 human cells across eight samples (table S1). Through unsupervised clustering, seven tumor clusters (11,501 cells) and seven NK clusters (11,786 cells) were classified (Fig. 7A) on the basis of the respective canonical marker genes of mesothelioma (*MSLN*, *KRT8*, and *KRT18*) and NK cells [*PTPRC*, *NCAM1*, *NKG7*, and *hYP218* (CAR)] (Fig. 7B), as well as top marker genes (up to 100) expressed in each cluster (data S1).

### Up-regulation of MHC molecules and reduction of target *MSLN* in TGF-β–rich tumors post–MSLN.CAR-IL-15 iNK cell treatment

We next investigated the features of tumor subclusters. Consistent with the flow cytometry and immunofluorescence findings, we observed both tumor clusters and NK clusters as the major populations (57% NK cells versus 43% tumor cells) in the MSLN.CAR-IL-15 GR1.1-iNK–treated tumors as compared to the untreated tumors (Fig. 7C). Using copyKAT copy number variant analysis (*24*), we identified T2, T3, T4, and T6 as the malignant tumor clusters (Fig. 7D) with high expression of *MSLN* (Fig. 7B). We then focused on these clusters for the downstream analysis. The proportion of T3, which expressed the highest level of *MSLN* among all the tumor subclusters, was significantly reduced after MSLN.CAR-IL-15 GR1.1-iNK treatment (Fig. 7E). Instead, clusters T2 and T6 emerged as the dominant populations after treatment. The four malignant tumor clusters highly expressed mesenchymal genes and *TGFB1*, along with various genes linked to cell growth, metabolism, inflammation, and stress responses, highlighting tumor heterogeneity and the critical role of transforming growth factor–β (TGF-β) in creating an immunosuppressive microenvironment. In addition, we observed significant reduction of *MSLN* expression in all the four clusters in the treated group (Fig. 7G), indicating effective targeted specific killing mediated by MSLN.CAR-IL-15 iNK. Further analysis of Hallmark pathway enrichment revealed that allograft rejection, interferon-γ response, and interferon-α response pathways were enriched in all the tumor clusters, while tumor necrosis factor–α (TNF-α) signaling via NF-κB and epithelial mesenchymal transition pathways were distinctly enriched in different subclusters (Fig. 7H). The expression of both human lymphocyte antigen (HLA) class I– and HLA class II–related genes was significantly up-regulated in tumor clusters post-iNK treatment (Fig. 7I), which contributed to the allograft rejection pathway enrichment score, suggesting that tumor cells may regulate iNK cell activity through the major histocompatibility complex (MHC) molecules (*25*, *26*).

### Robust activation, increased cytotoxicity, and cycling ability of tumor-infiltrating MSLN.CAR-IL-15 iNK cells

**Fig. 8.**
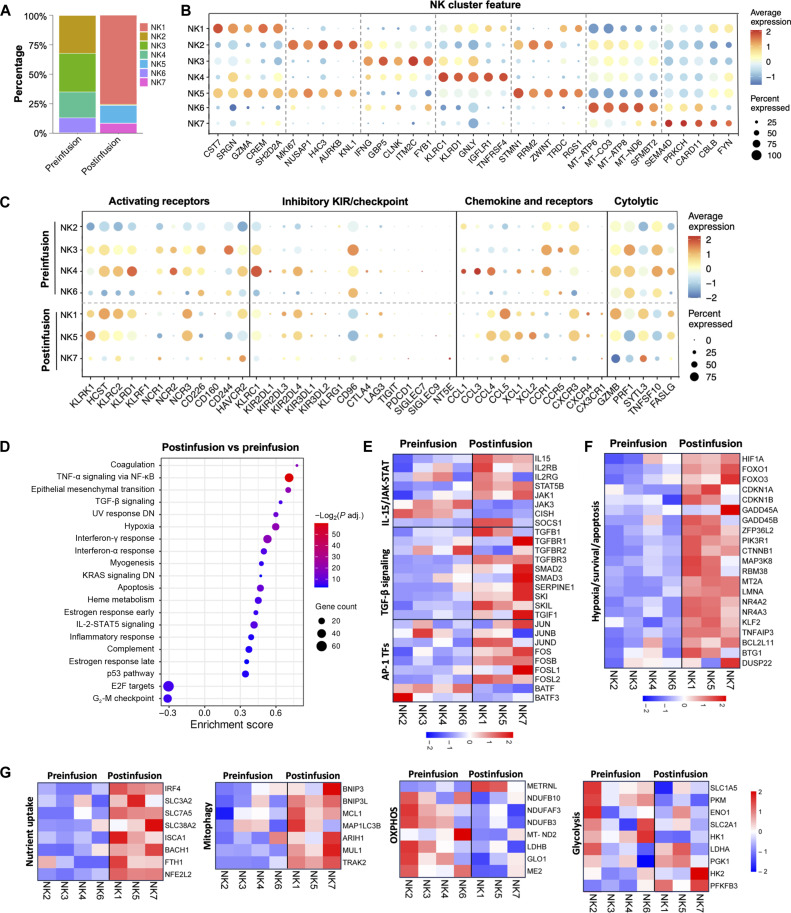
Single-cell transcriptional profiles of NCI-meso63 tumor–infiltrating CAR-IL-15 iNK cells. (**A**) Fractions of defined NK subclusters in NK cells in preinfusion and postinfusion groups. (**B**) Expression of representative signature genes across NK subclusters. (**C**) Expression of NK-associated biomarker genes (activation, inhibitory receptors, cytokines, chemokines, and cytolytic-related categories). (**D**) Up-regulated and down-regulated Hallmark pathways in total postinfusion versus preinfusion iNK cells (adjusted *P* < 0.1). (**E** to **G**) Heatmap indicating the expression of selected gene sets in NK subtypes.

Last, we sought to elucidate the alterations in the transcriptional profile of the iNK cells after they encounter the tumor microenvironment. The preinfusion iNK cells were mainly composed of 32.1% NK2, 32.7% NK3, 21.9% NK4, and 12.6% NK6, as compared to the tumor-infiltrating (post-infusion) iNK cells primarily composed of 75.4% NK1, 15% NK5, and 8.5% NK7 (Fig. 8A). The hYP218-scFv sequence (CAR) expression was observed to be higher in tumor-infiltrating NK1, NK5, and NK7 compared to preinfusion NK clusters, suggesting that the high expression of CAR may be essential for directing iNK cells into the tumor (Fig. 7B). We observed distinct characteristics of preinfusion and postinfusion iNK cells based on the expression of cluster-defining marker genes (Fig. 8B). In the preinfusion NK subclusters, NK2 consisted of highly proliferative iNK cells in culture, marked by high expression of cell division genes like *MKI67* and *AURKB*; NK3 represented activated NK cells, with high expression of *IFNG* and NK signaling genes such as *CLNK* and *FYB1*; the NK4 cluster showed elevated expression of inhibitory receptors *KLRC1*/*KLRD1* (NKG2A/CD94) and *TNFRSF4* (OX40); and NK6 displayed high levels of mitochondrial genes, indicating cell stress and cellular damage. In the postinfusion NK cells, NK1 was the dominant subcluster, expressing high levels of cytotoxic effector genes (*CST7*, *SRGN*, and *GZMA*), indicating its key role in tumor cell killing. The NK5 cluster was characterized by high expression of cell division genes, including *STMN1*, *RRM2*, and *ZWINT*, along with the tumor-infiltrating NK marker *RGS1* (*27*), suggesting that they may correspond to the Ki67^+^ NK population observed in immunofluorescence imaging. In addition, NK5 coexpressed marker genes of both the NK1 and NK2 clusters, which are associated with proliferation and cytotoxicity, highlighting its strong cytolytic activity and ability to persist in the tumor environment. NK7, marked by high expression of *SEMA4D* (CD100), *PRKCH* (PKCθ), and *CBLB*, exhibited the lowest expression of NK function–related genes among the postinfusion clusters. Notably, *RGS1*, *CBLB*, and *FYN* were expressed at higher levels, while *IFNG* was expressed at lower levels across all tumor-infiltrating NK clusters compared to the preinfusion NK clusters.

We next analyzed the expression of NK-associated biomarkers in the NK subclusters (Fig. 8C). Tumor-infiltrating clusters NK1 and NK5 showed high expression of NK-activating receptors, including NKG2D-DAP10 (*KLRK1*/*HCST*), NKG2C (*KLRC2*), CD94 (*KLRD1*), and NKp30 (*NCR3*), suggesting that they were robustly activated. Among the inhibitory killer cell immunoglobulin-like receptor (KIR) and immunosuppressive checkpoint genes, we observed high expression of *KIR2DL4*, intermediate levels of NKG2A (*KLRC1*), and notable reduction of *CD96* in the tumor-infiltrating iNK cells, along with negligible expression of other checkpoint markers. The protein expression of cell surface biomarkers of NKG2D, NKG2C, CD94, NKp30, NKG2A, and KIR2DL4 in tumor-infiltrating CAR-IL-15 iNK cells was validated through flow cytometry (fig. S6). The chemokine and receptor profiles of tumor-infiltrating NK1 and NK5 showed increased expression of *CCL5*, *CCL4*, *XCL1*, *XCL2*, and *CXCR3*, suggesting that they may be involved in NK tumor homing and activation (*28*, *29*). In contrast, *CCR1* and *CCR5* were decreased in tumor-infiltrating NK cells, which promote NK liver homing (*30*, *31*). Regarding cytotoxicity-related genes, *GZMB* and *SYTL3* were up-regulated in the NK1 cluster, while *PFR1* (perforin) and *TNFSF10* (TRAIL) were down-regulated in tumor-infiltrating iNK cells as compared to preinfusion iNK cells.

### Reduction of *CISH*, *TGFBR2*, and *BATF* in IL-15–producing tumor-infiltrating iNK cells to adapt to the hypoxic, TGF-β–rich tumor microenvironment

The pathway enrichment analysis revealed the pathways significantly enriched in tumor-infiltrating NK cells versus preinfusion NK cells, including TNF-α signaling via NF-κB, hypoxia, interferon-γ response, interferon-α response, apoptosis, heme metabolism, IL-2-STAT5 (signal transducers and activators of transcription 5) signaling, estrogen response, and p53 pathway (Fig. 8D). In contrast, the cell division–related pathway E2F target and G_2_-M checkpoint were down-regulated in the tumor-infiltrating NK cells compared to the highly proliferative preinfusion NK product. These findings indicate that tumor-infiltrating NK cells were actively engaged in complex biological processes to mount an effective immune response against the tumor and tune their own function and metabolism to adapt to the tumor microenvironment. We then analyzed the expression of different gene sets to understand the biological processes across different NK subclusters.

We observed significant up-regulation of IL-15 signaling genes in tumor-infiltrating NK clusters, including *IL15*, *IL2RB*, *IL2RG*, *STAT5B*, and *JAK1* (Fig. 8E). Notably, these genes exhibited higher expression levels in the NK1 subcluster compared to NK5. Unexpectedly, *JAK3* was down-regulated in postinfusion NK cells compared to preinfusion iNK cells. In addition, the negative regulators of IL-15 signaling *CISH* were markedly reduced, while *SOCS1* was markedly elevated in postinfusion iNK cells as compared to preinfusion iNK cells. In the tumor microenvironment characterized by high levels of TGF-β, NK1 and NK5 clusters exhibited low expression of *TGFBR2* (with particularly low levels in the NK5 cluster) and simultaneously up-regulated negative regulators of TGF-β signaling, *SKI*, *SKIL*, and *TGIF1*, indicating that these NK clusters may be adapting to mitigate the suppressive effects of TGF-β. In contrast, the cluster NK7 with an impaired NK function showed high expression of genes involved in TGF-β signaling. We also noted that activator protein 1 family transcription factors were enriched in postinfusion NK clusters except for the gene *BATF*, which exhibited lower expression in postinfusion NK cells compared to preinfusion NK cells.

A series of genes related to cell cycle arrest, DNA repair, and cell survival processes was enriched in postinfusion NK1 and NK5 clusters at similar levels (Fig. 8F), contributing to NK response to hypoxia and collectively enabling NK cells to thrive in challenging tumor microenvironments. The pro-apoptotic genes *BCL2L11*, *BTG1*, and *DUSP22* were expressed at lower levels in NK5 compared to NK1. As compared to preinfusion NK cells, postinfusion NK cells showed up-regulation of genes involved in nutrient uptake and mitophagy, lower levels of oxidative phosphorylation (OXPHOS), and similar levels of glycolysis (Fig. 8G). Enhanced nutrient uptake in tumor-infiltrating NK suggests that NK cells are adapting to their environment by increasing the availability of amino acids and other substrates, which can support cell survival and function despite other metabolic impairments (*32*). Up-regulation of mitophagy may help remove dysfunctional mitochondria, thereby maintaining mitochondrial health and function, even when overall oxidative metabolism is compromised (*33*). This may help NK cells maintain their effector functions. We also observed high expression of *METRNL* in postinfusion NK1 and NK5, which may be involved in disrupting the mitochondrial function in NK cells (*34*). In addition, NK5 displayed higher levels of OXPHOS and glycolysis compared to NK1, aligning with the increased proliferation observed in NK5. This metabolic fitness advantage in NK5 may enable these NK cells to meet the metabolic demands necessary for both cytotoxicity and proliferation, thereby enhancing their sustained functionality in the hypoxia-stressed tumor microenvironment.

## DISCUSSION

In this study, we identified LiPSC-GR1.1 as a superior iPSC cell line for producing an iNK product using a standardized simple differentiation method. Through genetic modification of LiPSC-GR1.1 with an MSLN-targeting CAR-IL-15 construct, we achieved robust iNK differentiation yield. We also demonstrated the characteristics and functionality of these MSLN.CAR-IL-15 iNK cells through comprehensive in vitro and in vivo experiments, including validating CAR expression and profiling iNK cell biomarkers, and demonstrated the tumor killing efficacy of these engineered iNK cells against MSLN-positive solid tumors. In addition, we observed a significant infiltration of MSLN.CAR-IL-15–engineered NK cells into MSLN-positive tumor, demonstrating their tumor homing ability and proliferation within the tumor microenvironment. Last, we detailed the single-cell transcriptional profiles of iNK-treated and untreated tumors, as well as of tumor-infiltrating MSLN.CAR-IL-15 iNK cells, which uncovered the early responses of MSLN-positive tumor cells to iNK cell–mediated killing and provided a comprehensive analysis of characteristics of MSLN.CAR-IL-15 iNK cells adapting to the solid tumor context.

iPSC-NK differentiation is a prolonged and intricate process (*35*). To address the challenges of large-scale production, enrichment and expansion of CD34^+^ hematopoietic progenitor cells before iNK differentiation are frequently used, with further postdifferentiation NK cell expansion using feeder cells being a necessary step (*36*). The spin EB method developed by Zhu and Kaufman (*21*) to generate iPSC-NK under feeder-free conditions without CD34^+^ enrichment significantly simplifies the operational workflow. The drawback is that only certain iPSC lines can be efficiently differentiated into NK cells using this method and the iNK yield is relatively low. Here, we identified LiPSC-GR1.1 as a superior source for producing iNK using this simple method. By introducing IL-15 in LiPSC-GR1.1, we increased the iPSC-NK differentiation yield by nearly 10-fold, which could significantly shorten the expansion and simplify the manufacturing process. While IL-15 engineering alone is expected to increase iNK yield and enhance NK cell activity, CAR engineering likely further augments the selective and sustained cytotoxicity of IL-15–expressing iNK cells against tumor cells, as demonstrated by the superior antitumor efficacy of CAR-IL-15–expressing cord blood NK cells compared to those expressing IL-15 alone (*37*). IL-15 plays a multifaceted role in the regulation of NK cells. While it is essential for NK cell development and homeostasis, prolonged exposure to IL-15 can result in NK cell exhaustion (*38*). Of note, our dosing experiment indicated that higher IL-15 concentrations did not necessarily result in better differentiation outcomes. We observed that increasing IL-15 to a high level (e.g., 80 ng/ml) had a detrimental impact on NK cell differentiation. Therefore, picking iPSC colonies highly expressing IL-15 for NK production may negatively affect subsequent NK cell differentiation. We also demonstrate that freshly differentiated MSLN.CAR-IL-15 GR1.1-iNK cells can be cryopreserved as a stock and subsequently expanded after thawing to an even larger scale without loss of functionality. This approach offers a standardized method for streamlined iNK production using the LiPSC-GR1.1 cell line. In addition, this protocol can be adapted for various CAR genetic modifications or strategies to enhance CAR NK therapy.

The MSLN.CAR-IL-15 iNK product expresses most activating NK receptors as well as the inhibitory receptors NKG2A and CD96. Upon entry into tumors, MSLN.CAR-IL-15 iNK cells showed increased NKG2D and NKp30 expression, together with decreased CD96 and NKG2A expression, but maintained a high level of KIR2DL4. Since HLA-G expression was also observed to be up-regulated in tumors, it will be important to test whether blockade of HLA-G/KIR2DL4 signaling can further improve the vulnerability of the tumors to iNK treatment. Other well-known checkpoint molecules that suppress the NK cell function, such as TIGIT, CTLA-4, KLRG1, and PD-1 (*39*), were found to be negative in MSLN.CAR-IL-15 iNK cells, even after infiltrating and interacting with tumors, suggesting that the GR1.1-NK product has a superior ability to counteract these suppressive mechanisms developed by tumors.

Strategies targeting *CISH* and TGFBR2 have been explored to enhance NK cell therapies. *CISH* knockout reprograms iPSC-derived NK cell metabolism, increases their sensitivity to IL-15 signaling, and improves in vivo persistence in tumor models (*40*). In addition, disrupting the TGF-β pathway through TGFBR2 knockout has been shown to enhance NK cell–mediated tumor killing (*41*, *42*). While targeting the TGF-β pathway can help prevent NK cell dysfunction, once the dysfunction has been established, it becomes persistent because of significant epigenetic reprogramming. It is reported that IL-15 could impair TGF-β signaling in human T lymphocytes (*43*). It is likely that the endogenous IL-15 expression in CAR iNK cells helped counteract TGF-β signaling–induced suppression on iNK cells in the tumor. A recent study revealed that the activator protein 1 transcription factor BATF plays a crucial role in driving epigenetic reprogramming triggered by the TGF-β pathway. This reprogramming leads to the up-regulation of checkpoint molecules such as CTLA-4, LAG3, PD-1, and NT5E, resulting in NK exhaustion (*44*). We found that MSLN.CAR-IL-15 iNK cells express very low level of *CISH*, *TGFBR2*, and *BATF* when infiltrating tumors. This unique profile may favor NK cells to effectively navigate the tumor microenvironment, thus optimizing their antitumor responses while mitigating the risks of exhaustion.

It is observed that *BTG1* was highly expressed in the tumor-infiltrating cluster NK1, with an intermediate level expressed in NK5. Recently, a phase 1 trial study of GD2-CAR-IL-15 NKT in refractory neuroblastoma showed that *BTG1* is a key driver of hyporesponsiveness in exhausted NKT and T cells (*45*). Knockdown of *BTG1* in GD2-CAR-IL-15 NKT cells enhanced tumor elimination in a neuroblastoma mouse mode. Thus, we hypothesize that deletion of *BTG1* may also enhance iNK antitumor efficacy. In addition, the *DUSP22* level was found to be significantly lower in the cycling NK5 cluster compared to NK1, with the lowest expression also observed in the highly proliferative preinfusion NK2 cluster. Previous studies have shown that DUSP22 inhibits T cell expansion by inducing apoptosis, whereas its knockout promotes T cell activation (*46*, *47*). Therefore, *DUSP22* could serve as another potential target for future research aimed at improving iNK persistence.

Despite the enhanced antitumor activity of MSLN.CAR-IL-15 iNK cells observed in different tumor models, tumor relapse eventually occurred even with multiple administrations of the product. MSLN.CAR-IL-15 iNK mediated specific killing against MSLN-positive tumor cells and led to the selective expansion of MSLN^low^ tumor cells, highlighting that antigen loss is always a concern for CAR-directed cellular therapy (*48*). In addition, tumors responded to NK treatment by expressing TGF-β, up-regulating MHC I/II molecules and genes involved in the interferon-γ response and TNF-α signaling via NF-κB pathways. While MHC I can directly limit NK cell activation via inhibitory receptors, MHC II can create a regulatory network that suppresses NK cell activity and promotes tumor immune evasion (*49*, *50*). At the same time, such MHC molecule up-regulation on tumor cells may enhance tumor recognition by T cells. A combination therapy study has shown that iPSC-NK helped recruit T cells to the tumor site and cooperated with T cells to exert enhanced tumor killing (*36*). NK cells may regulate a range of other immune cells, including dendritic cells and macrophages (*51*). IL-15 produced by MSLN.CAR-IL-15 iNK cells may also enhance the survival and functional capabilities of tumor-resident NK cells and CD8^+^ T cells (*52*). Future experiments using the constantly improving humanized mouse models may help address the cross-talk between NK cells and other immune cells in the tumor microenvironment.

In summary, we report a proof-of-concept approach for robustly differentiating iNK from the LiPSC-GR1.1 iPSC line genetically modified to stably express MSLN.CAR-IL-15. We demonstrate the substantial antitumor effects of these iNK cells against solid tumors, conduct a deep characterization of their phenotypic and transcriptional states, and report how the interplay between tumor cells and MSLN.CAR-IL-15 iNK cells affects both populations during therapy. Our work supports the clinical translation of MSLN.CAR-IL-15–engineered GR1.1-iNK for the treatment of patients with advanced treatment refractory solid tumors that highly express MSLN.

## MATERIALS AND METHODS

### Study design

The goals of this study included the development of a robustly differentiated MSLN-targeting CAR-IL-15 iNK product using the cGMP-manufactured iPSC line LiPSC-GR1.1 to phenotypically characterize MSLN.CAR-IL-15 GR1.1-iNK and to investigate the antitumor efficacy and the mechanism of action of MSLN.CAR-IL-15 GR1.1-iNK cells in MSLN-positive solid tumors. We tested six iPSC lines for iNK differentiation using the spin EB method. LiPSC-GR1.1 was identified as a superior iPSC line for iNK differentiation and selected for genetic modification using an MSLN.CAR-IL-15–expressing PiggyBac transposon/transposase system we created. LiPSC-GR1.1 iPSCs stably expressing MSLN.CAR-IL-15 were differentiated to GR1.1-iNK cells. We then determined NK cell receptor expression levels on GR1.1-iNK cells by flow cytometry and analyzed the transcriptome of GR1.1-iNK cells compared to PBNK cells isolated from healthy donors using bulk RNA-seq. The antitumor efficacy of MSLN.CAR-IL-15 GR1.1-iNK against MSLN-positive solid tumors was evaluated using in vitro and mouse tumor models. In addition, we investigated the infiltration of MSLN.CAR-IL-15 GR1.1-iNK in solid tumor tissues by flow cytometry and multiplex immunofluorescence. Last, to better understand tumor responses to MSLN.CAR-IL-15 GR1.1-iNK, as well as NK signatures associated with tumor homing and antitumor effects, we performed scRNA-seq analysis on the total cells isolated from MSLN.CAR-IL-15 GR1.1-iNK–treated tumor tissues, untreated tumor tissues, and preinfusion MSLN.CAR-IL-15 GR1.1-iNK product.

### Mice, tumor cell lines, iPSC lines, and reagents

NOD/SCID/γc^−/−^ (NSG) mice were obtained from the Jackson Laboratory (Bar Harbor, ME). All mice were maintained in a dedicated pathogen-free environment following NIH guidelines. Animal procedures reported in this study were conducted under an animal study protocol (no. TGOB-001) approved by the by the Animal Care and Use Committee of the National Cancer Institute and in accordance with federal regulatory requirements and standards. Established tumor cell lines KLM-1 MSLN-WT/KO [received from C. Alewine, CCR, National Cancer Institute (NCI), NIH, Bethesda, MD], OVCAR-8 (received from H. Kobayashi, CCR, NCI, NIH, Bethesda, MD), N87 (obtained from American Type Culture Collection, Manassas, VA), and the EBV-transformed B cell line SMI-LCL (received from R. W. Childs, Cellular and Molecular Therapeutics Branch, National Heart Lung and Blood Institute, NIH, Bethesda, MD) (*53*) were maintained in RPMI 1640 medium with 10% fetal bovine serum (FBS), 2 mM l-glutamine, and 100 U penicillin-streptomycin. Early-passage mesothelioma cell lines (NCI-meso21, NCI-meso29, and NCI-meso63) were maintained in RPMI 1640 medium with 20% FBS, 2 mM l-glutamine, and 100 U penicillin-streptomycin. The mesothelioma cell lines were established from ascites or pleural fluid obtained from patients with mesothelioma treated at the NCI (Bethesda, MD) under an Institutional Review Board–approved protocol (ClinicalTrials.gov NCT 01950572). The methods for the establishment of primary culture cell lines have been described previously (*54*). KLM-1 MSLN-WT/KO, OVCAR-8, NCI-meso29, and NCI-meso63 were stable luciferase-expressing cell lines (*12*). The iPSC line LiPSC-GR1.1 was generated at Lonza Walkersville Inc., as previously described (*9*, *10*), and distributed through RUCDR Infinite Biologics at Rutgers University (https://stemcells.nindsgenetics.org/). The iPSC lines NCRM1, NCRM2, NCRM4, NCRM5, and NCRM6 were obtained from the iPSC core of the National Heart, Lung, and Blood Institute (NHLBI). These lines are available to the research community. All the iPSC lines were generated from healthy donor–derived cord blood CD34^+^ cells and cryopreserved in CryoStor CS10 (STEMCELL Technologies). Recombinant human cytokines used in NK differentiation and culture included VEGF and BMP-4 (both obtained from R&D Systems Inc.), as well as SCF, IL-3, IL-15, IL-7, Flt3 ligand, IL-2, and IL-21 (all obtained from PeproTech). The antibodies used for flow cytometry and immunofluorescence are included in table S2.

### CAR construct cloning

Lentiviral packaging plasmid psPAX2 expressing CD8.BB^ICD^.Z.CAR (composed of MSLN-targeted hYP218 scFv, CD8a hinge spacer, CD8 TM, 4-1BB costimulatory ICD, and stimulatory domain CD3ζ chain), as well as a truncated human EGFR polypeptide (EGFRt), was created previously (*12*). The fragment of IL-15 plus T2A between EGFRt and IL-15 was synthesized as a gene fragment (by GeneWiz) and subcloned into the psPAX2 vector through seamless cloning using the NEBuilder HiFi DNA Assembly Cloning Kit (New England Biolabs) according to the manufacturer’s instructions. CD8 TM and 4-1BB-ICD were then replaced with a gene fragment of NKG2D TM and 2B4-ICD (synthesized by Genescript) through seamless cloning to generate plasmid psPAX2 expressing NKG2D/2B4^ICD^.Z.CAR as well as NKG2D/2B4^ICD^.Z.CAR-IL15. The PiggyBac transposon vector PBCAG-eGFP with a CAG promoter was a gift from J. Loturco (Addgene plasmid no. 40973) (*55*). To create CAR-expressing PBCAG plasmids, the expression cassette encoding NKG2D/2B4^ICD^.Z.CAR-EGFRt or NKG2D/2B4^ICD^.Z.CAR-EGFRt-IL-15 was amplified from the template plasmid psPAX2 through polymerase chain reaction and then subcloned into the Xma I and Not I sites of the PBCAG vector through seamless cloning.

### Cell culture of human iPSCs

iPSC lines were maintained feeder free in complete Essential 8 Flex medium (Thermo Fisher Scientific) in six-well plates coated with truncated vitronectin recombinant human protein (Thermo Fisher Scientific, A14700) at a concentration of 0.5 μg/cm^2^. They were routinely passaged as small clumps using 0.5 mM EDTA in phosphate-buffered saline (PBS) at a split ratio of 1:6 to 1:10 every 3 to 4 days after reaching 60 to 80% confluence. After EDTA treatment, hiPSCs were transferred to new vitronectin-coated plates in fresh medium supplemented with the ROCK inhibitor (ROCKi) Y-27632 (10 μM, R&D Systems Inc.). The next day, the medium was changed to hiPSC medium without ROCKi.

### NK cell differentiation from human iPSCs

The derivation of NK cells from iPSCs has been previously described (*21*). Briefly, 8000 TrypLE-adapted iPSCs were seeded in 96-well round-bottom plates with APEL medium containing SCF (40 ng/ml), VEGF (20 ng/ml), BMP-4 (20 ng/ml), and Y-27632 (10 μM). After 6 days of hematopoietic differentiation, spin EBs were washed once with NK differentiation medium and then transferred into six-well plates with NK differentiation medium containing IL-3 (5 ng/ml; first week only), IL-15 (10 ng/ml), IL-7 (20 ng/ml), SCF (20 ng/ml), and Flt3 ligand (10 ng/ml) for 4 weeks. Half-medium changes were performed twice a week. NK cells were harvested 4 weeks post-NK differentiation. For the IL-15 concentration test, different concentrations of IL-15 (10, 20, 40, and 80 ng/ml) were used during 4 weeks of NK differentiation. Three independent experiments were performed.

### Stable CAR constructs expression in the LiPSC-GR1.1 line

LiPSC-GR1.1 cells were dissociated as a single-cell suspension using TrypLE and then washed with fresh E8 medium supplemented with ROCKi. A total of 0.2 million iPSCs was harvested through centrifugation, resuspended in 20 μl of P3 primary solution containing 1 μg of CAR-expressing PBCAG transposon vector plus 0.25 μg of Super PiggyBac Transposase (cat. no. PB210PA-1, System Bioscience), and transferred into nucleocuvette strips. Nucleofection was performed using Lonza 4D-Nucleofector with pulse setting CA-137 according to the manufacturer’s guidelines. The iPSCs nucleofected without PBCAG transposon were used as mock control. CAR-expressing iPSCs were enriched through FACS of EGFR^+^ iPSCs during days 7 to 9 postnucleofection.

### IL-15 ELISA

iPSCs were seeded at 3 × 10^5^ cells per well in a six-well plate and cultured for 72 hours without a medium change. Then, the supernatants of the iPSC culture were collected. IL-15 was analyzed using the ELISA MAX Deluxe Set Human (BioLegend) according to the manufacturer’s guidelines. Three independent experiments were performed.

### iNK cryopreservation and expansion

Freshly differentiated iNK cells were harvested 4 weeks postdifferentiation and cryopreserved in CryoStor CS10 at 1.0 × 10^7^ cells/ml. For expansion testing, iNK cells were initially thawed and cultured in complete Xpander media supplemented with 10% heat-inactivated human AB serum, IL-2 (100 U/ml), IL-21 (100 ng/ml), and irradiated [100 gray (Gy)] SMI-LCL feeder cells (7.5:1 feeder-to-NK cell ratio) with a starting cell concentration of 1.0 × 10^6^ cells/ml. Expanded iNK cells were harvested on day 7 postexpansion for the following studies.

### PBNK isolation

Human peripheral blood mononuclear cells (PBMCs) from healthy donors were obtained from the NIH Clinical Center Department of Transfusion Medicine under the NIH Institutional Review Board–approved and NIH Institutional Review Board–consented healthy donor program. PBMCs were isolated by density gradient centrifugation on Histopaque 1077 (Sigma-Aldrich). Briefly, 15 ml of the collected blood sample was diluted in an equal volume of PBS and layered over onto an equal volume of Histopaque 1077 (Sigma-Aldrich). Gradients were centrifuged at 400*g* for 30 min at room temperature. The PBMC interface was carefully removed by pipetting and washed twice with PBS by centrifugation at 300*g* for 10 min. For the cell isolation, the cell number was determined using a Countess Automated Cell Counter (Invitrogen). Primary NK cells were purified from PBMCs using the human NK Cell Isolation Kit (Miltenyi Biotec). The NK purity was confirmed as 80 to 95% by flow cytometry. Three independent experiments were performed.

### In vitro NK cell cytotoxicity assays

NK cell cytotoxicity was determined in vitro in a coculture of NK cells and tumor cells by measuring direct killing of MSLN-expressing tumor cells by the NK cells. Briefly, 100 μl of complete RPMI 1640 media containing 5000 tumor cells was seeded in a 96-well U-bottom plate. Four hours after seeding the tumor cells, 100 μl of media containing NK cells was added into the coculture across a range of effector-to-target (E:T) ratios. The luciferase signal from the remaining live tumor cells was determined 24 hours postcoculture using the Luciferase Assay System (Promega, catalog no. E1501). Percent killing was calculated using the following formula: % Killing = 100 × [1 − Relative light units (RLUs) from coculture wells/RLU from target-alone wells]. Two or three independent experiments were performed.

### Mouse tumor model study

To evaluate iNK cell function against NCI-meso63 tumor in vivo, 5- to 6-week-old female NSG mice (the Jackson Laboratory) were intraperitoneally injected with 3 × 10^6^ luciferase-expressing NCI-meso63 tumor cells on day −7. BLI (PerkinElmer, IVIS Lumina III In vivo Imaging System) was performed on day −1. The mice were then conditioned with 225-cGy radiation and divided into groups with similar starting BLI values. The two doses of 8 × 10^6^ iNK cells (harvested either on day 28 post-NK differentiation or 1 week postexpansion from frozen iNK stock) were intraperitoneally injected into tumor-bearing mice on days 0 and 7. Tumor growth was monitored weekly via BLI. To evaluate the iNK cell function against KLM-1 tumor cells in vivo, NSG mice were intraperitoneally injected with 5 × 10^5^ luciferase-expressing KLM-1 tumor cells on day −5. BLI imaging was performed on day −1. The mice were then conditioned with 225-cGy radiation and randomly divided into groups with similar starting BLI values. Two doses of 1 × 10^7^ and one dose of 5 × 10^6^ iNK cells harvested during days 28 to 35 post-NK differentiation were intraperitoneally injected into tumor-bearing mice on days 0 and 7, and tumor growth was monitored weekly via BLI (*n* = 5 to 7 mice per group for the tumor model efficacy study). To investigate the tumor response and iNK cell tumor infiltration in the NCI-meso63 tumor model, we injected the mice with 6 × 10^6^ NCI-meso63 tumor cells on day −16 and treated the mice with 6 × 10^6^ iNK cells (product of 1-week expansion from frozen iNK stock) on day 0. BLI was performed to monitor the tumor growth on days −1 and 6. All the tissues and blood were harvested on day 7 post-iNK treatment. The tumors were harvested for flow cytometry, immunofluorescence, and scRNA-seq analysis. The spleens were harvested for flow cytometry analysis (n = 3 mice per group). Two or three independent experiments were performed.

### Bulk mRNA sequencing

Total RNA was isolated from freshly isolated PBNK cells (from healthy donors) and freshly differentiated iNK cells harvested 4 weeks post-NK differentiation using TRIzol reagent according to the manufacturer’s guide (Invitrogen). The quantity and quality of RNA were analyzed using Agilent TapeStation. mRNA sequencing samples were pooled and sequenced on NextSeq 2000 P2 using the Illumina stranded mRNA ligation kit having 77 to 88 million pass filter reads with more than 92% of bases above the quality score of Q30. Reads of the samples were trimmed for adapter quality bases using Cutadapt before alignment with the reference genome (hg38) and the annotated transcripts using STAR. The average mapping rate of all samples was 95%. Unique alignment was above 90%. There were 3.33 to 4.82% unmapped reads. The mapping statistics were calculated using Picard software. The samples had 0.47% ribosomal bases. Percent coding bases were between 42 and 51%. Percent untranslated region bases were 31 to 33%, and mRNA bases were between 74 and 83% for all the samples. Library complexity was measured in terms of unique fragments in the mapped reads using Picard’s MarkDuplicate utility. The samples had 70 to 75% nonduplicate reads. In addition, gene expression quantification analysis was performed for all samples using STAR/RSEM tools. The normalization of the RNA counts and DEG analysis were performed using the Limma Voom R package. Transcripts with significantly differential expression (*P* < 0.05, log_2_ fold change >1) were used for subsequent pre-ranked GSEA using the EasyGSEA tool (*56*) for Hallmark and KEGG gene set scoring.

### Single-cell isolation from mouse tissues

From NCI-meso63 tumor–bearing mice, spleens were harvested in cold PBS, gently mashed, and filtered through a two-chamber sterile filter bag (Thermo Fisher Scientific), and the resulting single-cell suspension was collected from the other side of the bag. The mouse peritoneal tumors were harvested in cold serum-free RPMI 1640 medium, and total cells were isolated using a tumor dissociation kit (Miltenyi Biotec) according to the manufacturer’s instructions. Briefly, tumor tissues were digested in serum-free RPMI 1640 medium supplemented with human tumor dissociation kit enzymes (Miltenyi Biotec) at 37°C under continuous rotation for 90 min. Dissociated single-cell suspensions were then filtered through a 70-μm mesh filter and pelleted at 300*g* for 5 min. For flow cytometry analysis, all the single cells isolated from the spleens and tumors were washed with FACS buffer (PBS + 0.5% FBS + 1 mM EDTA). For scRNA-seq study, the single cells isolated from the tumors were washed with PBS + 0.04% bovine serum albumin (BSA). The single cells prepared were then used for the following study.

### Flow cytometry

For human iPSCs, iPSCs were dissociated into a single-cell suspension with TrypLE, harvested, washed, then resuspended in E8 medium containing Essential 8 Flex medium, and incubated with fluorescent conjugated antibodies at 4°C for 30 min. For iNK cells, cells were harvested from the cultured supernatant, washed, then resuspended in PBS, and incubated with fluorescent conjugated antibodies at 4°C for 30 min. For the cells harvested from NK/tumor coculture or isolated from peritoneal tumors and spleens, cells were first stained with the Zombie Aqua Fixable Viability Kit (BioLegend, cat. no. 423102) at room temperature for 30 min and then incubated with 1% FBS at 4°C for 15 min to block nonspecific binding sites before incubating with fluorescent conjugated antibodies at 4°C for 30 min. For CD107a degranulation assay, NK cells were incubated with or without KLM-1 cells at 2:1 E:T ratios in the presence of an anti-CD107a antibody for 1 hour, followed by addition of GolgiStop (BD Biosciences, cat. no. 554724) for an additional 2 hours of incubation. At the completion of incubation, cells were washed with PBS, then first stained with the Zombie Aqua Fixable Viability Kit at room temperature for 30 min, and then incubated with 1% FBS at 4°C for 15 min to block nonspecific binding sites before incubating with fluorescent conjugated antibodies at 4°C for 30 min. Flow cytometry detection was performed on CytoFLEX LX and analyzed using FlowJo software. Two or three independent experiments were performed.

### Multiplex immunofluorescence

Fresh isolated tumor tissues were fixed in BD Cytofix/Cytoperm solution (BD Bioscience, cat. no. 554722) diluted 1:4 in PBS for 2 days at 4°C, washed three times in PBS, and maintained in PBS for 10 hours at 4°C. Then, the tissue blocks were transferred into PBS containing 30% sucrose for 2 days until the tissue blocks sank to the bottom of the 30% sucrose solutions. The tissues were then embedded in Scigen Tissue-Plus O.C.T. Compound (Thermo Fisher Scientific, cat. no. 23-730-571) and stored at −80°C before use. Cross sections (12-μm-thick) of the tumor tissue blocks were cut by using a Leica CM1950 cryostat. The O.C.T. compound of tissue sections was removed by washing the tissue sections with PBS for 30 min before being blocked in 1% BSA and 0.1 M tris buffer (pH 7.4) containing 0.3% Triton X-100 for 30 min at room temperature. A microwave-assisted immunofluorescence staining protocol was adapted for MSLN detection by using a mouse anti-human MSLN antibody amplified by a fluorescent dye–directly conjugated donkey anti-mouse secondary antibody (*57*). The sections were then blocked in 1% BSA and 1% mouse serum in 0.1 M tris buffer (pH 7.4) containing 0.3% Triton X-100 and stained with the following fluorescent dye–directly conjugated primary antibodies: CD45-AF532, CD56-PE/CF594, and Ki67-AF700 together with nuclear dye Helix NP-NIR (BioLegend, cat. no. 425301). Then, tissue sections were washed in 0.1 M tris buffer (pH 7.4) and mounted with SlowFade Gold Antifade Mounting solution (Thermo Fisher Scientific, cat. no. S36937) using no. 1.5 cover glass (VWR, cat. no. 48393-241). Digital scan of stained tumor sections was performed using an inverted Leica TCS SP8 X confocal system equipped with an 80-MHz pulsed white light laser, four gallium-arsenide Hybrid Detectors, and one multialkali photomultiplier tube with spectral detection capability. A 40× (numerical aperture, 1.3) oil emersion objective lens was used for scanning the sections with a pixel size of 568.74 mm by 568.74 mm and a pixel dwell time of 1.2 ms. Digital images acquired by the Leica confocal system were tile stitched and then processed to correct signal spillovers from nearby channels and the autofluorescence signal within the tumor sections by using the manual unmixing method of the Automatic Dye Separation function within the Leica Application Suite X (LAS X) software package (version 4.4.0.24861). The output images (.lif files, Leica file format) were converted into Imaris version 5.5 files by Imaris software (version 9.5.0, Bitplane) and imported into Imaris. Images of individual channels were passing through a Gaussian filter to remove random noise before pseudocolor assignment and visualization. In all image processing steps, the image size and aspect ratio were maintained identical by keeping the pixel size unchanged. Two or three independent experiments were performed.

### Single-cell RNA sequencing

Eight single-cell samples were prepared for scRNA-seq, including two samples of preinfusion product, three samples of total cells isolated from the untreated tumors (individual mouse as one sample), and three samples of total cells isolated from the MSLN.CAR-IL-15 iNK–treated tumors (four mice pooled as one sample). Dead cells and debris were removed using the Dead Cell Removal Kit (Miltenyi Biotec, 130-090-101) following the manufacturer’s instructions. Single-cell suspensions were washed once with ice-cold PBS with 0.04% BSA by centrifugation at 350*g* and gently resuspended in fresh buffer. The cellular samples were loaded in the lane according to the 10x Genomics Chromium Next GEM Single Cell 5′ v2 (Dual Index) user guide with a single capture lane per sample. The recovery was 6000 cells per lane. Cell partitioning completed with a uniform emulsion consistency, and the reverse transcription polymerase chain reaction was run. All subsequent steps of library preparation and quality control were performed as described in the 10x Genomics user guide. Then, the sequencing was performed on the NovaSeq 6000 system with the NovaSeq S2 Reagent kit version 1.5 (100 cycles) following the cycling parameters: 26 base pairs (bp; read1), 10 bp (index 1), 10 bp (index2), and 90 bp (read2).

### scRNA-seq data processing

Base calling was performed using RTA 3.4.4, demultiplexing was performed using CellRanger version 8.0.0 (Bcl2fastq 2.20.0), and alignment was performed using cellranger version 8.0.0 (STAR 2.7.2a). Sequenced reads were aligned to a custom human GRCh38 and mouse GRCm39 reference sequence made by adding the hYP218-scFv sequence to the refdata-gex-GRCh38_and_GRCm39-2024-A reference. Unique molecular identifier (UMI)–adjusted aligned reads were used to generate a single-cell barcode and gene expression matrix for downstream analysis.

The preprocessed gene expression matrix generated by the CellRanger (10x Genomics) pipeline was imported into Seurat (version 5.1.0). Putative droplet doublets were detected using scDblFinder (version 1.16.0) with default parameters. Doublet cells were removed from each sample separately. Samples were merged. As a quality control step, all cells expressing <200 genes were removed, as well as cells that contained <500 UMIs and >20% mitochondrial counts. The filtered gene expression matrix was normalized using the NormalizeData function with default parameters. A total of 3000 variable genes was identified using the FindVariableFeatures function with the vst method. PCA was applied with the RunPCA function to reduce dimensionality after regressing for the number of UMIs (counts) and percentage mitochondrial genes with the ScaleData function. The 15 most informative principal components were used for clustering and uniform manifold approximation and projection (UMAP) for dimension reduction. Shared nearest neighbors were computed with the FindNeighbors function, and cells were then clustered using the FindClusters function with the resolution set to 0.8. A UMAP was generated with the RunUMAP function. After the first round of unsupervised clustering, we calculated the percentages of human and mouse genes in a cell in each cell cluster. The mouse cell clusters were removed from downstream analysis, which focused on human cells. Genes of the mouse genome were also removed from human cells. Then, we performed a second round of unsupervised clustering on human cells. The second-round clustering procedure was performed the same as the first-round clustering with a 0.7 resolution and dimensionality reduction with top 20 principal components for visualization. Human NK cells and tumor clusters were annotated using curated in-house signatures. The cells annotated as B cells (LCL NK feeder cells) were removed from the analysis. R (version 4.3.1) and RStudio (2023.09.1 Build 494, “Desert Sunflower” Release) were used for analysis. DEGs were performed using the FindMarkers function with the Wilcoxon rank sum test, with the logfc.threshold, min.pct, and only.pos parameters set to log_2_(1.5), 0.1, and F, respectively. DEGs were filtered with a cutoff of either pct.1 or min pct.2 > 0.25 and adjusted *P* < 0.05 for GSEA. Gene set variation analysis implemented in the fgsea R package (version 1.28.0) was used for GSEA with 10,000 permutations performed. The Hallmark gene sets were retrieved from the msigdbr R package (version 7.5.1). The input genes were calculated by FindMarkers and sorted by avg_log2FC, and enriched pathways with less than three genes in leadingEdge or adjusted *P* > 0.25 were filtered out. To recognize malignant cell subsets, we used copyKAT (version 1.1.0) to determine human normal cells and malignant cells with the hg20 genome and other parameters set as default (*24*). The identified aneuploid cells were extracted as malignant cells.

### Statistical analysis

Unpaired Student’s *t* test was used to compare the differences between groups for in vitro and in vivo studies. Mouse survival differences were compared using the log-rank (Mantel-Cox) test. Statistical analysis was performed using GraphPad Prism 6.0. *P* < 0.05 was considered statistically significant.
