# Supplementary material for: Robust differentiation of NK cells from MSLN.CAR-IL-15–engineered human iPSCs with enhanced antitumor efficacy against solid tumors
Source: Sci Adv. 2025 May 2;11(18):eadt9932. doi: 10.1126/sciadv.adt9932 (PMC12047432; doi:10.1126/sciadv.adt9932)
Supplement: Supplementary file 1 — Supplementary Materials and Methods Figs. S1 to S6 Tables S1 and S2 Legend for data S1 [file sciadv.adt9932_sm.pdf]

Science Advances
AAAS

Supplementary Materials for

Robust differentiation of NK cells from MSLN.CAR-IL-15–engineered human iPSCs with enhanced antitumor efficacy against solid tumors

Qun Jiang *et al.*

Corresponding author: Qun Jiang, jiangq2@mail.nih.gov; Raffit Hassan, hassanr@mail.nih.gov

Sci. Adv. **11**, eadt9932 (2025)
DOI: 10.1126/sciadv.adt9932

The PDF file includes:

Supplementary Materials and Methods
Figs. S1 to S6
Tables S1 and S2
Legend for data S1

Other Supplementary Material for this manuscript includes the following:

Data S1

Materials and Methods

Trilineage Differentiation and Scorecard Assay

Directed three germ layer differentiation was performed using StemMACS™ Trilineage Differentiation Kit (Miltenyi Biotec, 130-115-660) according to manufacturer's instructions. The cells were harvested, and RNA was extracted using the PureLink™ RNA Mini Kit (Thermo Fisher Scientific, 12183018A). DNase-treated RNA was prepared according to the High-capacity cDNA Reverse Transcription kit with RNase Inhibitor (Thermo Fisher Scientific, 4374966). The differentiation potential was assessed by quantitative PCR using the hPSC Scorecard Assay (Thermo Fisher Scientific, A16179) according to the manufacturer’s instructions. The scores representing gene expression associated with self-renewal, ectoderm, mesoderm or endoderm tissues were calculated according to algorithms determined by the manufacturer relative to a reference standard.

Sample Name	Self-renewal	Ectoderm	Mesoderm	Endoderm
NCRM1	-4.29	1.31	4.94	1.17
NCRM2	-4.19	1.33	4.14	1.05
NCRM4	-4.56	1.32	4.13	1.53
NCRM5	-4.78	1.01	4.93	1.17
NCRM6	-4.25	0.69	4.48	1.18

Gene expression relative to the reference standard

Upregulated						Downregulated
x > 1.5	1.0 < x ≤ 1.5	0.5 < x ≤ 1.0	-0.5 ≤ x ≤ 0.5	-1.0 ≤ x < -0.5	-1.5 ≤ x < -1.0	x < -1.5

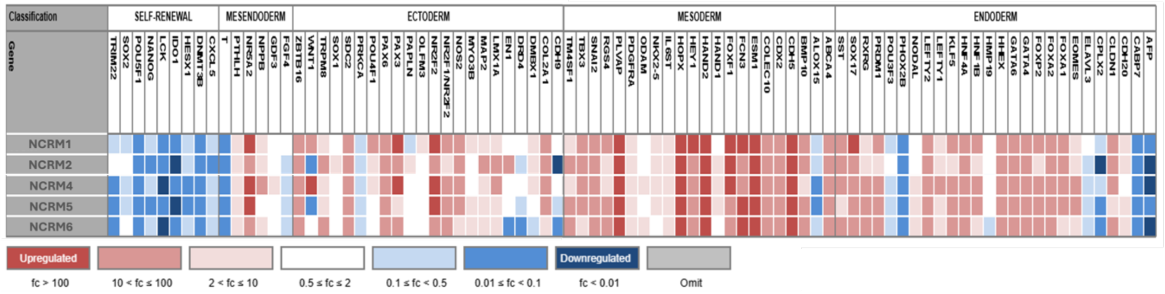

Fig. S1. Tri-lineage differentiation ability of the iPSC lines. The Scorecard assay confirms pluripotency and differentiation potential of the iPSC line NCRM1, NCRM2, NCRM4, NCRM5, and NCRM6. Top panel showed Scorecard Values. Algorithm scores for the samples show up regulation or down regulation of the endoderm, mesoderm, ectoderm or pluripotent (self-renewal) markers relative to the reference set of nine undifferentiated pluripotent stem cell lines. Bottom panel is the heatmap of gene expression associated with self-renewal, mesendoderm, ectoderm, mesoderm and endoderm. Colors correlate to the fold change in expression of the indicated gene relative to the undifferentiated reference set.

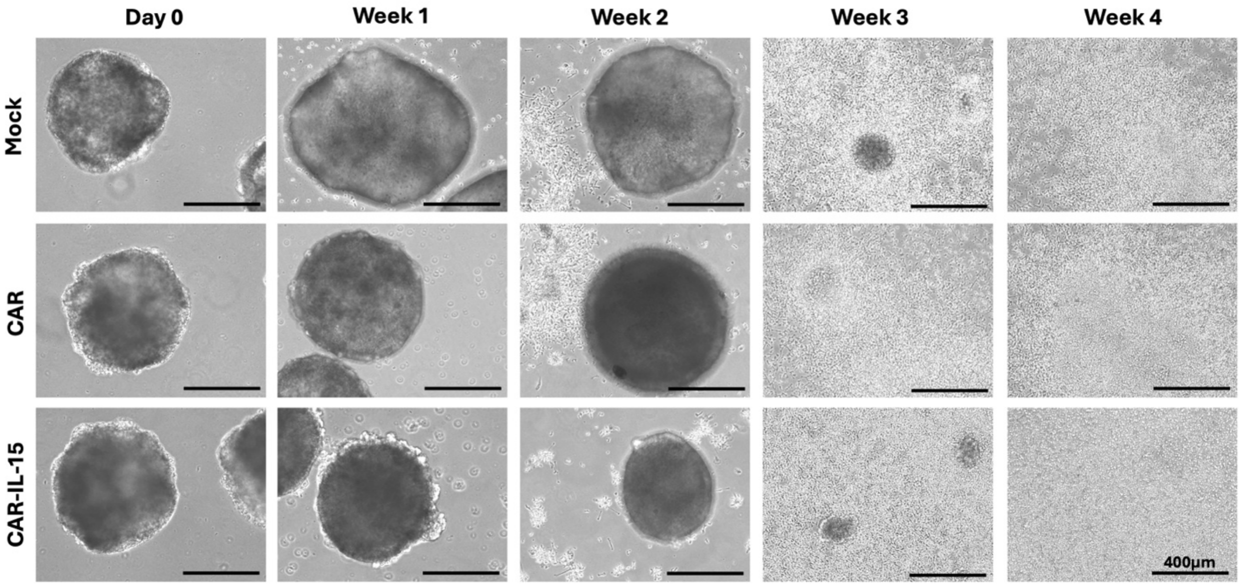

Fig. S2. The representative pictures of iNK differentiation from genetically modified or mock iPSCs cell line LiPSC-GR1.1. day 0 EBs and 4 weeks iNK differentiation from mock, MSLN.CAR-expressing, and MSLN.CAR-IL-15 expressing iPSC were shown. The bars represent 400µm.

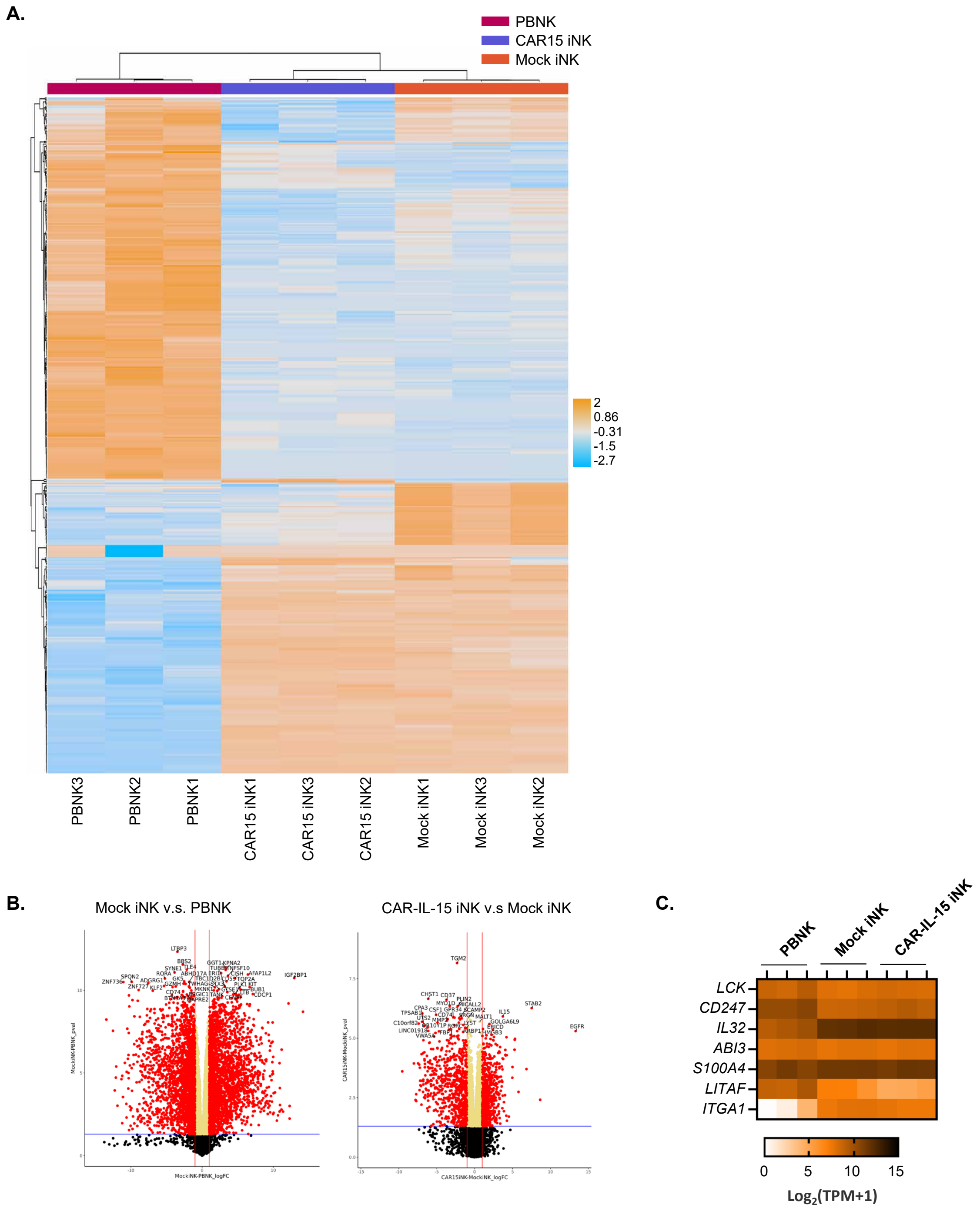

Fig. S3. Differentially expressed genes analyses of RNA-seq data of different NK products. PBNKs were purified from 3 healthy donors. mock iNKs and MSLN.CAR-IL-15 iNKs were harvested 4 weeks post iNK differentiation. (A) Heatmap of hierarchical clustering of differentially expressed genes. (B) Volcano plot of log2 (fold change) and –log10FDR of all tested genes between mock iNK v.s. PBNK, as well as between MSLN.CAR-IL-15 iNKs v.s. mock iNKs. Each dot in red represents an individual gene differentially expressed with

log2FC >1, $p < 0.05$. (C) The heatmap shows the gene expression (log2 (TPM+1)) of “adaptive” NK markers in PBNKs, mock iNKs, and MSLN.CAR-IL-15 iNKs.

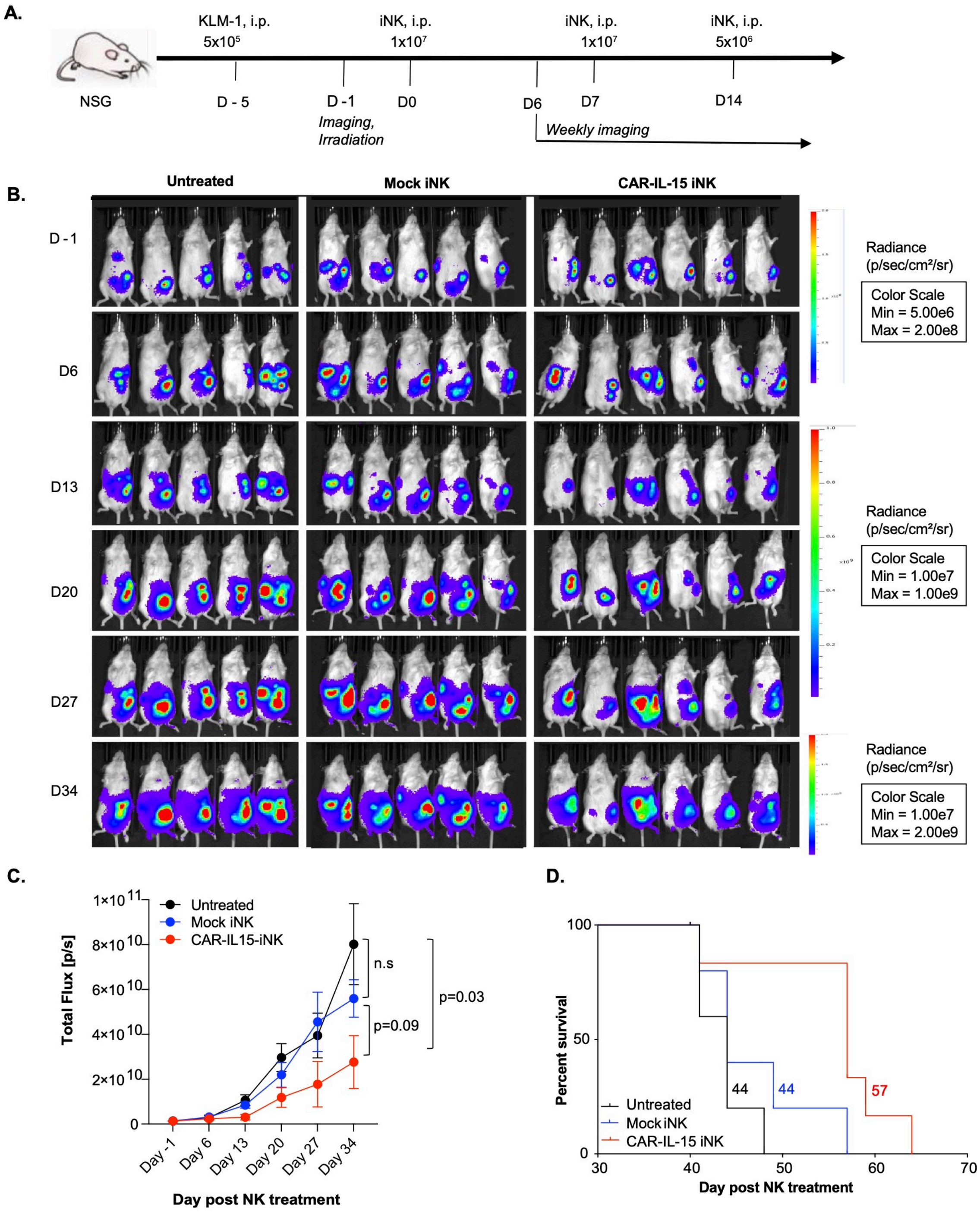

Fig. S4. In vivo efficacy of freshly differentiated GR1.1-iNK cells against human KLM-1 pancreatic tumors. (A) Schematic of tumor inoculation and iNK treatments in KLM-1 tumor model. KLM-1 cells were i.p. injected 5 days before iNK treatment. The mice were then administrated three doses of freshly differentiated iNKs on day 0, day 7 and day 14. (B) Tumor growth monitored through BLI. (C) Summarized tumor growth curve is shown. (D) The median overall survival of different groups post iNK treatment is shown ($p<0.05$, CAR-IL-15 iNK vs Mock iNK).

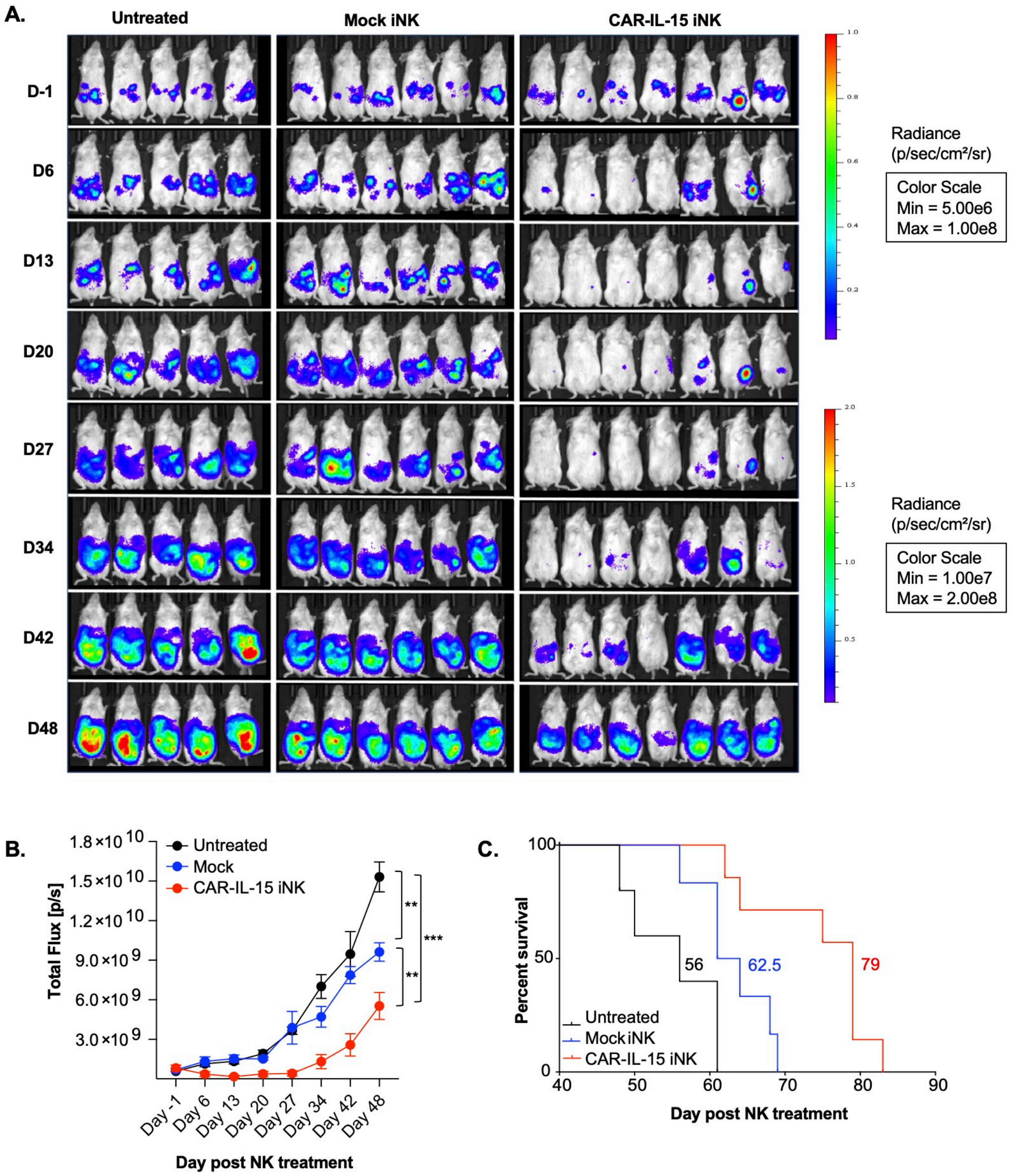

Fig. S5. In vivo efficacy of expanded frozen GR1.1-iNK cells against NCI-meso63 tumors. (A) Tumor growth monitored through BLI. (B) Summarized tumor growth curve is shown. (C) The median overall survival of different groups post iNK treatment is shown ($p<0.05$, Mock iNK vs Untreated; $p=0.01$, CAR-IL-15 iNK vs Mock iNK).

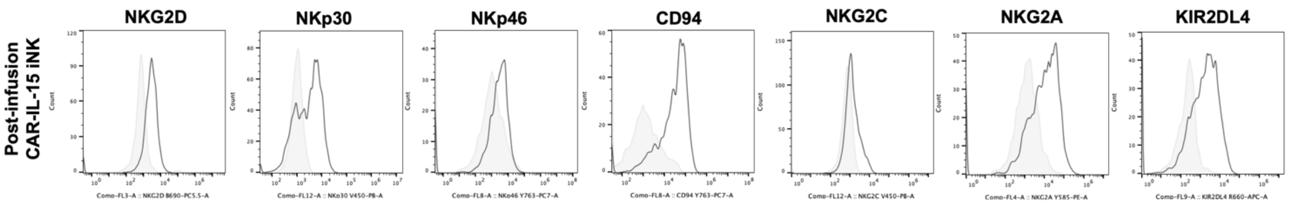

Fig. S6. The surface biomarker expression of post-infusion CAR-IL-15 iNKs. Flow cytometry was performed to analyze the iNKs in tumors on day 7 post CAR-IL-15 iNKs treatment. $hCD56^+$ cells were gated to analyze the indicated biomarkers expression. The solid open lines represent biomarker staining. The grey area represents isotype control.

Table S1. scRNAseq subcluster cell number.

Clusters	Cell number	Untreated group	Treated group	Pre-infusion NK
T1	4159	3027	1132	
T2	1723	466	1257	
T3	1668	1406	262	
T4	995	656	339	
T5	755	526	229	
T6	1635	884	751	
T7	566	394	172	
**Total tumor**	**11501**	**7359**	**4142**	
NK1	4270	1	4260	9
NK2	2011	1	41	1969
NK3	2006	0	2	2004
NK4	1354	0	10	1344
NK5	852	1	851	0
NK6	782	2	7	773
NK7	511	4	478	29
**Total NK**	**11786**	**9**	**5649**	**6128**
**Total cells**	**23287**			

Table S2. Antibodies used in Flow cytometry and multiplex immunofluorescence.

Flow Cytometry Antibodies	Source
Mouse anti-human CD56 -BV785, Clone: 5.1H11	BioLegend, Cat#362549
Rabbit anti-human EGFR-APC, Clone: C225 (Cetuximab)	Novus Biologicals, Cat#NBP2-52671APC
Mouse anti-human DNAM-1-PerCP/Cy5.5, Clone: 11A8	BioLegend, Cat#338313
Mouse anti-human NKp46-PE/Cy7, Clone: 9E2	BioLegend, Cat#331915
Mouse anti-human NKG2D-FITC, Clone: 1D11	BioLegend, Cat#320820
Mouse anti-human TRAIL-PE/Cy7, Clone: RIK-2	BioLegend, Cat#308216
Mouse anti-human Fas-L-PE, Clone: NOK-1	BioLegend, Cat#306407
Mouse anti-human CD16-PerCP/Cy5.5, Clone: B73.1	BioLegend, Cat#360712
Mouse anti-human NKp44-PerCP/Cy5.5, Clone: P44-8	BioLegend, Cat#325113
Mouse anti-human CD94-PE/Cy7, Clone: DX22	BioLegend, Cat#305515
Mouse anti-human NKG2A-PE, Clone: 131411	R & D Systems, Cat# FAB1059P-025
Mouse anti-human CD158 (KIR2DL1/S1/S3/S5)-FITC, Clone: HP-MA4	BioLegend, Cat#339504
Mouse anti-human CD107a-PerCP/Cy5.5, Clone: H4A3	BioLegend, Cat#328616
Mouse anti-human CD45-PE-Cy7, Clone: HI30	BioLegend, Cat#982310
Rat anti-mouse CD45-PerCP/Cy5.5, Clone: 30-F11	BioLegend, Cat#103132
Mouse anti-human CD3- BV510, clone: OKT3	BioLegend, Cat#317331
Mouse anti-human CD158d (KIR2DL4)-APC, Clone: mAb 33	BioLegend, Cat#347007
Mouse anti-human CD159c (NKG2C)-BV421, Clone: S19005E	BioLegend, Cat#375013
Mouse anti-anti-human CD337 (NKp30)-BV421, Clone: P30-15	BioLegend, Cat#325227

Immunofluorescence Antibodies	Source
Mouse anti-human CD45-AF532, Clone: HI30	Thermo Fisher Scientific, Cat# 58-0459-41
Mouse anti-human CD56-PE-CF594, Clone: B159	BD Biosciences, Cat# 562289
Mouse anti-human Ki67-AF700, Clone: B56	BD Biosciences, Cat# 561277
Mouse anti-human Mesothelin, Clone: MN-1	Rockland, Cat# 200-301-A88
Donkey anti-mouse IgG-AF790	Thermo Fisher Scientific, Cat# A11371

Other Supplementary Materials for this manuscript include the following:

Data S1: Excel file containing top 100 marker genes for each subcluster analysis from CAR-IL-15 iNK-infiltrating tumor scRNA-seq dataset.
